# Mixed‐type multivariate response regression with covariance estimation

**DOI:** 10.1002/sim.9383

**Published:** 2022-03-24

**Authors:** Karl Oskar Ekvall, Aaron J. Molstad

**Affiliations:** ^1^ Division of Biostatistics, Institute of Environmental Medicine Karolinska Institutet Stockholm Sweden; ^2^ Applied Statistics Research Unit, Institute of Statistics and Mathematical Methods in Economics TU Wien Vienna Austria; ^3^ Department of Statistics and Genetics Institute University of Florida Gainesville Florida USA

**Keywords:** covariance estimation, latent variable models, mixed‐type response regression, multivariate regression

## Abstract

We propose a new method for multivariate response regression and covariance estimation when elements of the response vector are of mixed types, for example some continuous and some discrete. Our method is based on a model which assumes the observable mixed‐type response vector is connected to a latent multivariate normal response linear regression through a link function. We explore the properties of this model and show its parameters are identifiable under reasonable conditions. We impose no parametric restrictions on the covariance of the latent normal other than positive definiteness, thereby avoiding assumptions about unobservable variables which can be difficult to verify in practice. To accommodate this generality, we propose a novel algorithm for approximate maximum likelihood estimation that works “off‐the‐shelf” with many different combinations of response types, and which scales well in the dimension of the response vector. Our method typically gives better predictions and parameter estimates than fitting separate models for the different response types and allows for approximate likelihood ratio testing of relevant hypotheses such as independence of responses. The usefulness of the proposed method is illustrated in simulations; and one biomedical and one genomic data example.

## 1 INTRODUCTION

In many regression applications, there are multiple response variables of mixed types. For instance, when modeling complex biological processes like fertility (see Section 6.1), the outcome (ability to conceive) is often best characterized through a collection of variables of mixed types: both count (number of egg cells and number of embryos) and continuous (square‐root estradiol levels and log gonadotropin levels). Similarly, one may be interested in the dependence between various types of omic data in a particular genomic region (see Section 6.2), some binary (eg, presence of somatic mutations) and some continuous (eg, gene expression). Joint modeling of responses facilitates inference on their dependence, and it can lead to more efficient estimation, better prediction, and allows for the testing of joint hypotheses without the need for multiple testing corrections. Popular regression models, however, typically assume all responses are of the same type. A case in point is the multivariate normal linear regression model which assumes a vector of responses $Y\in {\mathbb{R}}^{r}$ and vector of predictors $x\in {\mathbb{R}}^{p}$ satisfy

(1) $$Y∼N_{r}(B^{T}x,\sum ),$$

for some regression coefficient matrix $B\in {\mathbb{R}}^{p\times r}$ and covariance matrix $\sum \in {𝕊}_{++}^{r}$, the set of r×r symmetric and positive definite matrices. Model (1) is fundamental in multivariate statistics and leads to relatively straightforward likelihood‐based inference; the parameters are identifiable, have intuitive interpretations, and maximum likelihood estimates are computationally tractable even when r and p are relatively large (but smaller than the number of independent observations n). Here, our goal is the development of a likelihood‐based method for mixed‐type response regression that retains some of the useful properties of (1). We focus on settings where dependence between responses cannot be parsimoniously parameterized and interest is in inference on an unstructured r×r covariance matrix characterizing this dependence. Rather than a method for specific combinations of response distributions, of which there are many,^1^, ^2^, ^3^, ^4^, ^5^, ^6^, ^7^, ^8^, ^9^, ^10^, ^11^ we pursue a unified method that can be adapted to many different combinations of response distributions with relative ease.

To develop our method, we assume there is a known link function $g:{\mathbb{R}}^{r}\to {\mathbb{R}}^{r}$ and latent vector $W\in {\mathbb{R}}^{r}$ such that

(2) $$g\{𝔼(Y\;|\;W)\}=W\;\;\text{and}\;\;W∼N_{r}(B^{T}x,\sum ).$$

That is, a latent vector satisfies the multivariate linear regression model and the observable responses are connected to that latent vector through their conditional mean. We further suppose that, conditionally on W, the elements of Y satisfy generalized linear models with linear predictors given by the elements of W (see detailed specification in Section 2). This leads to a likelihood based on n independent observations $\left\{(y_{i},x_{i})\in {\mathbb{R}}^{r}\times {\mathbb{R}}^{p},i=1,\ldots ,n\right\}$ which can be written

(3) $$L_{n}(B,\sum )=\overset{n}{\underset{i=1}{\prod }}\int_{{\mathbb{R}}^{r}}f(y_{i}\;|\;w_{i})\;\phi (w_{i};B^{T}x_{i},\sum )\;dw_{i},$$

where $f(y_{i}\;|\;w_{i})$ is the conditional density for $Y_{i}={\left[Y_{i1},\ldots ,Y_{ir}\right]}^{T}$ given $W_{i}={\left[W_{i1},\ldots ,W_{ir}\right]}^{T}$ and $\phi (\cdot ;B^{T}x_{i},\sum )$ is the multivariate normal density with mean $B^{T}x_{i}$ and covariance matrix ∑. Note (1) is a special case of (2) where Y=W and g is the identity. Conversely, (3) can be obtained as the likelihood for a specific generalized linear latent and mixed model (GLLAMM),^12^ a class of models which also includes many other models for mixed‐type responses as special cases.^13^, ^14^, ^15^, ^16^, ^17^, ^18^ Example 1 illustrates our setting.

Example 1 Suppose $Y\in {\mathbb{R}}^{20}$ is a vector of 10 count variables ($Y_{1},\ldots ,Y_{10}$) and 10 continuous variables ($Y_{11},\ldots ,Y_{20}$) whose joint distribution is of interest. If there are no predictors, a possible version of (2) assumes $Y_{j}|W∼\text{Poi}(W_{j})$ for j=1,…,10, $Y_{j}\;|\;W∼N(W_{j},1)$ for j=11,…,20, and $W∼N_{20}(B^{T},\sum )$, where $B^{T}\in {\mathbb{R}}^{20}$ and $\sum \in {𝕊}_{++}^{20}$.

There are several challenges with inference based on (3). First, in general the integrals cannot be computed analytically, and numerical integration can be prohibitively time consuming even for r≈10. This is commonly addressed by, for example, Laplace approximations, Monte Carlo integration, or penalized quasi likelihood (PQL), which results from dropping terms in a Laplace approximation that are assumed to be of lower order.^19^, ^20^, ^21^, ^22^, ^23^ Here, in the interest of developing a method operational for relatively large r, we will approximate the likelihood using a modified version of PQL (Section 3), which is known to be fast.^21^ We will also discuss how the proposed method can be extended to settings where the likelihood is approximated by other means.

A second challenge is that, even if the integrals in (3) can be computed efficiently, maximum likelihood estimation of (B,∑) requires optimization over ${\mathbb{R}}^{p\times r}\times {𝕊}_{++}^{r}$:

(4) $$(\hat{B},\hat{\sum })\in \underset{(B,\sum )\in {\mathbb{R}}^{p\times r}\times {𝕊}_{++}^{r}}{\text{arg max}}L_{n}(B,\sum ).$$

When r is small it may be possible to solve (4) using off‐the‐shelf optimizers (eg, the ml command in Stata or the optim function in R). For example, if r=2, then (4) can be characterized as a constrained optimization problem over $(B,{\left[\sum_{11},\sum_{12},\sum_{22}\right]}^{T})\in {\mathbb{R}}^{p\times r}\times {\mathbb{R}}^{3}$, the constraints being that $\sum_{11}>0$, $\sum_{22}>0$, and $\sum_{12}^{2}<\sum_{11}\sum_{22}$. When r is even moderately large, however, such constraints become untenable. In software packages for mixed and latent variable models it is common to maximize the likelihood in B and the Cholesky root L such that $\sum =LL^{T}$.^24^, ^25^ This ensures the estimate of ∑ is positive semidefinite, but it does not ensure positive definiteness and it complicates more ambitious inference. For example, implementing a likelihood‐ratio test of whether some of the responses are independent requires solving (4) subject to the constraint that some off‐diagonal elements of ∑ are zero (Section 4). Similarly, for some response‐types it is necessary to constrain diagonal elements of ∑ to ensure identifiability (see Section 2), and sometimes it is desirable to assume diagonal elements of ∑ corresponding to responses of the same type (eg, responses 1‐10 or 11‐20 in Example 1) are the same. These types of restrictions are nontrivial to accommodate when parameterizing in terms of L, but as we will see in Section 3, they are elegantly handled by the method we propose. Briefly, our method iteratively updates a PQL‐like approximation of (3) and maximizes that approximation in B and ∑ using block coordinate descent. The update for B is a least squares problem which can be solved in closed form and the update for ∑ is solved using an accelerated projected gradient descent algorithm. The algorithm scales well in the dimension r and it natively supports restrictions of the form ∑∈𝕄 for sets $𝕄\subseteq {𝕊}_{++}^{r}$ onto which a projection can be computed.

In some settings it may be appropriate to, as is common in mixed models, restrict $\sum =ZDZ^{T}$ for some design matrix $Z\in {\mathbb{R}}^{r\times d}$ and covariance matrix $D\in {𝕊}_{++}^{d}$ of d<r random effects (see eg, Jiang^26^). Such structures may be suggested by subject‐specific knowledge or the sampling design, or they may be motivated by necessity when n is small relative to r. Similarly, many methods based on the marginal moments, such as for example generalized estimating equations,^27^ specify a covariance structure. In many applications, however, it is unclear whether there is any particular dependence structure. Indeed, it may be of primary interest to discover a structure using data, rather than imposing one a priori; our method enables such discoveries. Some methods based on the marginal moments also allow the specification of an unstructured correlation matrix, but for Y rather than W.^28^, ^29^ In general, however, such methods are inconsistent with (2) since the mean vector and correlation matrix of Y cannot be independently parameterized. Accordingly, it is often unclear whether the estimates are in the parameter set of any particular model for mixed‐type responses. Nevertheless, methods based on the marginal moments can be useful in practice and they are a reasonable comparison to our method for some purposes.

Other advantages of the parameterization we consider are that (i) the off‐diagonal elements of ∑ affect the joint distribution of responses but not their marginal (univariate) distributions and (ii) responses are independent if the corresponding off‐diagonal elements of ∑ are zero. Thus, we can test the null hypothesis that some responses are independent by testing whether the corresponding off‐diagonal elements of ∑ are zero, without that null hypothesis implying restrictions on the marginal distributions. This is generally not possible in more parsimonious parameterizations of the form $\sum =ZDZ^{T}$ since, in general, elements of D affect both marginal and joint distributions.

## 2 MODEL

### 2.1 Specification

We now specify our model in detail and generalize somewhat compared to the Introduction. Assume the elements of $Y=(Y_{1},\ldots ,Y_{r})$ are conditionally independent given W with conditional densities of the form

(5) $$f(y_{j}\;|\;w)=\exp\left\{\frac{y_{j}w_{j}-c_{j}(w_{j})}{\psi_{j}}\right\},$$

where $\psi ={\left[\psi_{1},\ldots ,\psi_{r}\right]}^{T}$ is a vector of strictly positive dispersion parameters and $c_{j}$ is a cumulant function for the distribution of $Y_{j}\;|\;W$. For example, $c_{j}^{\prime }(W_{j})=𝔼(Y_{j}\;|\;W)$ and $\psi_{j}c_{j}^{\prime \prime }(W_{j})=\mathrm{var}(Y_{j}\;|\;W)$, with primes denoting derivatives. From these properties it follows that the link function g defined by g{𝔼(Y|W}=W satisfies $g(W)={\left[g_{1}(W_{1}),\ldots ,g_{r}(W_{r})\right]}^{T}$ with $g_{j}$ real‐valued and strictly increasing for j=1,…,r. That is, the jth latent variable has a direct effect on the jth response but no other responses. Equation (5) specifies a (conditional) generalized linear model^30^ (GLM), which when $\psi_{j}=1$ specializes to a one‐parameter exponential family distribution, as in Example 1. Here, we do not assume $\psi_{j}=1$ for every j but we do assume the $\psi_{j}$ are known.

Because it makes our development no more difficult, in what follows we consider a slightly more general version of (2) where

(6) $$W∼N_{r}(X\beta ,\sum ),$$

for a nonstochastic design matrix $X\in {\mathbb{R}}^{r\times q}$ and $\beta \in {\mathbb{R}}^{q}$. The classical multivariate response regression setting in (2) which motivates our study is then a special case with $X=I_{r}⊗x^{T}$, β=vec(B), and q=rp, where ⊗ is the Kronecker product and vec(·) the vectorization operator stacking the columns of its matrix argument. Unlike (2) where all responses have the same predictors, (6) allows distinct predictors for each response, as in seemingly unrelated regressions.^31^

With $\left\{(y_{i},X_{i})\in {\mathbb{R}}^{r}\times {\mathbb{R}}^{r\times p},i=1,\ldots ,n\right\}$, denoting independent realizations, the likelihood is

(7) $$L_{n}(\beta ,\sum )=|\sum {|}^{-n/2}\overset{n}{\underset{i=1}{\prod }}\int_{{\mathbb{R}}^{r}}\exp\left\{\left(\overset{r}{\underset{j=1}{\sum }}\frac{y_{ij}w_{ij}-c_{j}(w_{ij})}{\psi_{j}}\right)-\frac{1}{2}{\left(w_{i}-X_{i}\beta \right)}^{T}\sum^{-1}(y_{i}-X_{i}\beta )\right\}dw_{i}.$$

To see the connection to GLLAMMs and other mixed models which are often written for all observations simultaneously, let $𝒴={\left[Y_{1}^{T},\ldots ,Y_{n}^{T}\right]}^{T}\in {\mathbb{R}}^{rn}$, $𝒳={\left[X_{1}^{T},\ldots ,X_{n}^{T}\right]}^{T}\in {\mathbb{R}}^{rn\times q}$, and $ℰ∼N_{nr}(0,I_{n}⊗\sum )$. Then (7) is the likelihood for a model which assumes the elements of 𝒴 follow conditionally independent GLMs given ℰ, with canonical link functions and linear predictors given by the elements of

𝒲=𝒳β+ℰ;

that is, each of the rn responses has a linear predictor with its own random intercept, and the covariance matrix for those random intercepts is $I_{n}⊗\sum$.

### 2.2 Parameter interpretation and identifiability

It is often difficult to interpret parameters in latent variable models and, similarly, it is often unclear which parameters are identifiable—we address some such concerns in this section. The parameters are straightforward to interpret in the latent regression, but interpreting them in the marginal distribution of Y requires more work. To that end, note that the mean vector and covariance matrix of Y are, respectively, by iterated expectations,

(8) $$𝔼(Y)=𝔼\{g^{-1}(W)\}\;\;\text{and}\;\;\text{cov}(Y)=\text{cov}\{g^{-1}(W)\}+𝔼\{\text{cov}(Y\;|\;W)\}.$$

We make a number of observations based on (8): first, because cov(Y|W) is assumed diagonal, the covariance between responses is determined by $\text{cov}\{g^{-1}(W)\}$. Second, since $𝔼(Y_{j})$ and $𝔼(Y_{j}^{2})$ are determined by the univariate distribution of $Y_{j}$, off‐diagonal elements of ∑ do not affect means and variances of the responses. Third, since g and cov(Y|W) are nonlinear and nonconstant in general, 𝔼(Y) and 𝔼{cov(Y|W)} in general depend on both β and diagonal elements of ∑. Fourth, since $\mathrm{var}(Y_{j})$ is increasing in $\psi_{j}$ and $\text{cov}\{g^{-1}(W)\}$ does not depend on ψ, $\text{cor}(Y_{j},Y_{k})$ is decreasing in $\psi_{j}$ and $\psi_{k}$. This is intuitive as responses are conditionally uncorrelated and hence, loosely speaking, a large element of ψ indicates substantial variation in the corresponding response is independent of the variation in the other responses. In some settings, more precise statements are possible by analyzing closed form expressions for the moments in (8), as the next example illustrates.

Example 2 (Normal and Poisson responses) Suppose there are r=4 responses such that $𝔼(Y_{j}\;|\;W)=W_{j}$ and $\mathrm{var}(Y_{j}\;|\;W)=\psi_{j}$ for j=1,2, and $𝔼(Y_{j}|W)=\exp(W_{j})$ and $\mathrm{var}(Y_{j}\;|\;W)=\psi_{j}\exp(W_{j})$ for j=3,4. These moments are consistent with assuming $Y_{j}\;|\;W∼N(W_{j},\psi_{j})$ for j=1,2, and, if $\psi_{3}=\psi_{4}=1$, $Y_{j}|W∼\text{Poi}\{\exp(W_{j})\}$, j=3,4. When not assuming $\psi_{3}=\psi_{4}=1$, we say these moments are consistent with normal and (conditional) quasi‐Poisson distributions. We examine the effects of these assumptions on the marginal moments of Y. Some algebra (Supplementary Material) gives the following moments: $𝔼(Y_{1})=X_{1}^{T}\beta$, $𝔼(Y_{3})=\exp(X_{3}^{T}\beta +\sum_{33}/2)$, $\mathrm{var}(Y_{1})=\psi_{1}+\sum_{11}$, $\mathrm{var}(Y_{3})=\exp(2X_{3}^{T}\beta +\sum_{33})\{\exp(\sum_{33})-1+\psi_{3}\exp(-X_{3}^{T}\beta -\sum_{33}/2)\}$, $\text{cov}(Y_{1},Y_{2})=\sum_{21}$, $\text{cov}(Y_{1},Y_{3})=\sum_{31}\exp(X_{3}^{T}\beta +\sum_{33}/2)$, and $\text{cov}(Y_{3},Y_{4})=\exp(X_{3}^{T}\beta +X_{4}^{T}\beta +\sum_{33}/2+\sum_{44}/2)\{\exp(\sum_{43})-1\}$; the remaining entries of cov(Y) are the same as those given up to obvious changes in subscripts. Clearly, both 𝔼(Y) and cov(Y) depend on β and ∑, but regardless of type, the variance of $Y_{j}$ is increasing in $\sum_{jj}$, the mean is increasing in $X_{j}^{T}\beta$, and the covariance between $Y_{j}$ and $Y_{k}$ is increasing in $\sum_{jk}$. We will later use these observations to prove a result which implies β and ∑ are identifiable in this example.

Consider the linear dependence between responses with conditional normal and quasi‐Poisson moments, $Y_{1}$ and $Y_{3}$, say. The sign of their correlation is the sign of $\sum_{13}$ and the squared correlation satisfies, by Cauchy‐Schwarz's inequality, $\text{cor}{\left(Y_{1},Y_{3}\right)}^{2}\leq \sum_{11}\sum_{33}/[(\psi_{1}+\sum_{11})\{\exp(\sum_{33})-1+\psi_{3}/𝔼(Y_{3})\}]\leq \sum_{33}/\{\exp(\sum_{33})-1\}$. Thus, strong linear dependence between $Y_{1}$ and $Y_{3}$ requires a small $\sum_{33}$.

To gain intuition for how two responses with quasi‐Poisson moments behave, suppose for simplicity that $\sum_{33}=\sum_{44}$, $\psi_{3}=\psi_{4}$, and $X_{3}^{T}\beta =X_{4}^{T}\beta$. Then $\text{cor}(Y_{3},Y_{4})=\{\exp(\sum_{43})-1\}\{\exp(\sum_{33})-1+\psi_{3}/𝔼(Y_{3})\}$. For small $\psi_{3}$, this correlation is approximately $\left(\exp(\sum_{43})-1)/(\exp(\sum_{33})-1\right)$, which for $|\sum_{43}|\leq \sum_{33}$ is upper bounded by 1 and lower bounded by $\left\{\exp(-\sum_{33})-1\}/\{\exp(\sum_{33})-1\right\}$. The latter expression tends to −1 if $\sum_{33}\to 0$ and 0 if $\sum_{33}\to \infty$. Thus, strong negative correlation between $Y_{3}$ and $Y_{4}$ requires a small $\sum_{33}$.

Example 2 is convenient because the moments have closed form expressions. In more complicated settings, the following result can be useful. It implies the mean of $Y_{j}$ and covariance of $Y_{j}$ and $Y_{k}$ are strictly increasing in, respectively, the mean of $W_{j}$ and covariance between $W_{j}$ and $W_{k}$.

Lemma 1 *Let* $\phi_{\mu ,\sum }$ *be a bivariate normal density with marginal densities* $\phi_{\mu_{1},\sigma_{1}^{2}}$ *and* $\phi_{\mu_{2},\sigma_{2}^{2}}$ *and covariance* $\sigma =\sum_{12}=\sum_{21}$ *; then for any increasing, nonconstant* g,h:ℝ→ℝ *, the functions defined by* $\mu_{1}\mapsto \int g(t)\phi_{\mu_{1},\sigma_{1}^{2}}(t)\;dt$ *and* $\sigma \mapsto \int g(t_{1})h(t_{2})\phi_{\mu ,\sum }(t)\;dt$ *are, assuming the (Lebesgue) integrals exist, strictly increasing on* ℝ *and* $\left(-\sqrt{\sum_{11}\sum_{22}},\sqrt{\sum_{11}\sum_{22}}\right)$ *, respectively*.

The proof is in the Supplementary Material. We illustrate the usefulness of this result in another example.

Example 3 (Normal and Bernoulli responses) Suppose r=2 with $Y_{1}|W_{1}∼N(W_{1},\psi_{1})$ and $Y_{2}$ Bernoulli distributed with $𝔼(Y_{2}|W_{2})={\text{logit}}^{-1}(W_{2})$ $=1/\{1+\exp(-W_{2})\}$. Suppose also for simplicity $W∼N_{2}(\beta ,\sum )$, β∈ℝ2. The marginal distribution of $Y_{2}$ is Bernoulli with $𝔼(Y_{2})=\int [\phi (t)/\{1+\exp(-\beta_{2}-{\sqrt{\sum }}_{22}t)\}]\;dt,$ where ϕ(·) is the standard normal density. One can show that, if $\sum_{22}$ is fixed, $𝔼(Y_{2})\to 0$ if $\beta_{2}\to -\infty$ and $𝔼(Y_{2})\to 1$ if $\beta_{2}\to \infty$. That is, any success probability is attainable by varying β and, hence, some restrictions are needed for identifiability. One possibility, which has been used in similar settings, is to fix $\sum_{22}$ to some value, say one.^17^, ^32^ While fixing $\sum_{22}=1$ does not impose restrictions on the distribution of $Y_{2}$ as long as $\beta_{2}$ can vary freely, it may impose restrictions on the joint distribution of $Y={\left[Y_{1},Y_{2}\right]}^{T}$, properties of which we consider next.

Equation (8) implies $\text{cov}(Y_{1},Y_{2})=\int [\{t_{1}\phi_{\beta ,\sum }(t)\}/\{1+\exp(-t_{2})\}]\;dt-\beta_{1}𝔼(Y_{2})$. The integral does not admit a closed form expression, but Lemma 1 says the covariance is strictly increasing in $\sum_{12}$, which can be used to show the parameters are identifiable in this example if $\sum_{22}$ is known (Theorem 1). To understand which values $\text{cov}(Y_{1},Y_{2})$ can take, consider the limiting case as $\sum_{12}\to {\sqrt{\sum }}_{11}{\sqrt{\sum }}_{22}$ and assume for simplicity $\beta_{1}=\beta_{2}=0$. In the limit, the covariance matrix is singular and the distribution of W the same as that obtained by letting $W_{2}=\left(\sqrt{\sum_{22}}/\sqrt{\sum_{11}}\right)W_{1}$. Then one can show $\text{cor}(Y_{1},Y_{2})=2\int \left[\left\{\sqrt{\sum_{11}}t\phi (t)\right\}/\left\{1+\exp(-\sqrt{\sum_{22}}t)\right\}\right]\;dt/\sqrt{\psi_{1}+\sum_{11}}$. By using the dominated convergence theorem as $\psi_{1}\to 0$ and $\sum_{22}\to \infty$, this can be shown to tend to and be upper bounded by $\sqrt{2/\pi }\approx 0.8$ . This correlation corresponds to a limiting case and is an upper bound on the attainable correlation between Bernoulli and normal responses.

We conclude with a result on identifiability. The result is stated for some common choices of link functions and distributions of Y|W, but the proof can be adapted to other settings. In the Supplementary Material, we state a result (Lemma B.4) which outlines conditions for identifiability more generally. Essentially, when $Y_{j}\;|\;W$ satisfies a GLM, it suffices that, for every j, the variance of $Y_{j}$ is not a function of the mean of $Y_{j}$. If it is, as is the case when $Y_{j}$ is Bernoulli distributed, restrictions on diagonal elements of ∑ are needed for identifiability. The proof is in Supplementary Material.

Theorem 1 *Suppose* $\left\{(y_{i},X_{i})\in {\mathbb{R}}^{r}\times {\mathbb{R}}^{r}\times q;i=1,\ldots ,n\right\}$ *is an independent sample from our model and that (i)* $g_{j}$ *,* j=1,…,r *, is either the identity, natural logarithm, or logit (log‐odds) function; (ii)* $\sum_{jj}$ *is fixed and known for every* j *corresponding to logit* $g_{j}$ *; and (ii)* ${𝒳}^{T}𝒳$ *is invertible, then distinct* (β,∑) *correspond to distinct distributions for* 𝒴.

From a computational perspective, nonidentifiability can lead to likelihoods with infinitely many maximizers or ridges along which the likelihood is constant. In light of this result, we constrain the diagonals of ∑ corresponding to binary response variables: we found this leads to faster computation and improved estimation accuracy.

## 3 ESTIMATION

### 3.1 Overview

We propose an algorithm based on linearization of the conditional mean function $w\mapsto \nabla c(w)=g^{-1}(w)$, where $c(w)=\sum_{j=1}^{r}c_{j}(w_{j})$; this is essentially equivalent to linearization of the link function, which has been considered in other latent variable models.^19^ More specifically, consider the elementwise first order Taylor approximation of $g^{-1}(\cdot )=\nabla c(\cdot )$ around an arbitrary $w\in {\mathbb{R}}^{r}$: $𝔼(Y\;|\;W)=\nabla c(W)\approx \nabla c(w)+\nabla^{2}c(w)(W-w)$. Applying expectations and covariances on both sides yields $𝔼(Y)\approx \nabla c(w)+\nabla^{2}c(w)(X\beta -w)=:m(w,\beta )$ and $\text{cov}\{𝔼(Y\;|\;W)\}\approx \nabla^{2}c(w)\sum \nabla^{2}c(w)$. Approximating $𝔼\{\text{cov}(Y\;|\;W)\}=𝔼\{\text{diag}(\psi )\nabla^{2}c(W)\}\approx \text{diag}(\psi )\nabla^{2}c(w)$ leads to $\text{cov}(Y)\approx \text{diag}(\psi )\nabla^{2}c(w)+\nabla^{2}c(w)\sum \nabla^{2}c(w)=:C(w,\sum )$. Intuitively, we expect m(w,β) and C(w,∑) to be good approximations if W takes values near w with high probability. Now, consider a working model which says $Y_{1},\ldots ,Y_{n}$ are independent with

(9) $$Y_{i}∼N_{r}\{m(w_{i},\beta ),C(w_{i},\sum )\},$$

for observation‐specific approximation points $w_{i}\in {\mathbb{R}}^{r}$, i=1,…,n. The corresponding negative log‐likelihood is, up to scaling and additive constants

$$h_{n}(\beta ,\sum \;|\;w_{1},\ldots ,w_{n})=\overset{n}{\underset{i=1}{\sum }}\log det\left\{C(w_{i},\sum )\}+\overset{n}{\underset{i=1}{\sum }}{\left\{y_{i}-m(w_{i},\beta )\right\}}^{T}C{\left(w_{i},\sum \right)}^{-1}\{y_{i}-m(w_{i},\beta )\right\}.$$

If all responses are normal, then the working model is exact and minimizers of $h_{n}$ are maximum likelihood estimates (MLEs). More generally, minimizers of $h_{n}$ are approximate MLEs whose quality depend on the accuracy of the working model (9). For further insight it is helpful to note that if $w_{i}$ is set to the maximizer of the ith integrand in (7), then $h_{n}$ is the same type of approximation of $L_{n}$ as that used in PQL, specialized to our setting. The correspondence between linearization of the link function and PQL is detailed by Breslow.^22^ The correspondence implies that, in addition to the moment‐based motivation given here, $h_{n}$ can be motivated as a Laplace approximation of $L_{n}$, with terms assumed to be of lower stochastic order ignored (see Breslow and Clayton^20^ for details). Roughly speaking, the approximation will be better the closer the distribution of $Y_{i}$ is to a normal. Because the latent variables are normal, we expect the distribution of $Y_{i}$ to be close to normal if the distribution of $Y_{i}\;|\;W_{i}$ is. Now, to estimate variance parameters, conventional PQL makes further approximations which lead to a set of estimating equations. We proceed differently and avoid these approximations, both because they lack formal motivation (section 2.5 in Breslow and Clayton^20^) and because they do not in general lead to a simpler optimization problem for an unstructured and positive definite ∑. Thus, we will work directly with the approximate log‐likelihood $h_{n}$ and propose an algorithm that is substantially different from ones commonly used for mixed models.

With $h_{n}$ as the starting point, a natural algorithm for estimating β and ∑ would iterate between updating (β,∑) by minimizing $h_{n}$ with the $w_{i}$ held fixed and then updating the $w_{i}$ to get a more accurate working model. Motivated by the connection to Laplace approximations, we update the $w_{i}$ by setting them to equal to the maximizers of the integrand in (7) with β and ∑ fixed at their current iterates. To summarize, we propose a blockwise iterative algorithm whose (k+1)th iterates are obtained using the updating equations

(10) $$\begin{array}{ll} \left(\beta^{\left(k+1\right)},\sum^{\left(k+1\right)}\right) & =\underset{\beta ,\sum }{\text{arg min}}h_{n}(\beta ,\sum |w_{1}^{\left(k\right)},\ldots ,w_{n}^{\left(k\right)}), \end{array}$$

(11) $$\begin{array}{ll} \left(w_{1}^{\left(k+1\right)},\ldots ,w_{n}^{\left(k+1\right)}\right) & =\underset{w_{1},\ldots ,w_{n}}{\text{arg max}}\overset{n}{\underset{i=1}{\sum }}\log f_{\beta^{\left(k+1\right)},\sum^{\left(k+1\right)}}(y_{i},w_{i}). \end{array}$$

This algorithm can be run for a prespecified number of iterations or until convergence of the β and ∑ iterates, for example. While the complete algorithm is not designed to minimize a particular objective function, the individual updates, which we discuss in more detail shortly, minimize objective functions that can be tracked to determine convergence within each update. In our experience, the values of ∑ and β tend to converge after (at most) tens of iterations of (10) and (11). The final iterates of ∑ and β are approximate MLEs and $h_{n}$ evaluated at the final iterates of $w_{1},\ldots ,w_{n}$ is an approximate log‐likelihood which we will use for approximate likelihood‐based inference, including the construction of likelihood‐ratio tests.

A formal statement of the proposed algorithm is in Algorithm 1.

Algorithm 1 **Blockwise iterative algorithm for estimating (β,∑)
**

1

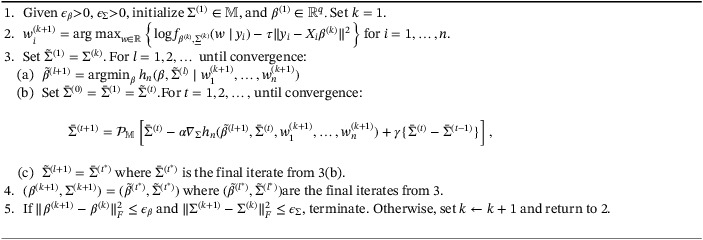

### 3.2 Updating β and ∑

To solve (10), we use a blockwise coordinate descent algorithm. Treating $w_{1},\ldots ,w_{n}$ as fixed throughout and ignoring the iterate superscript, this algorithm iterates between updating β and ∑. Specifically, the (l+1)th iterates of the algorithm for solving (10) can be expressed

(12) $$\begin{array}{ll} \beta^{\left(l+1\right)} & =\underset{\beta }{\text{arg min}}\;h_{n}\left(\beta ,\sum^{\left(l\right)}\;|\;w_{1},\ldots ,w_{n}\right), \end{array}$$

(13) $$\begin{array}{ll} \sum^{\left(l+1\right)} & =\underset{\sum }{\text{arg min}}\;h_{n}\left(\beta^{\left(l+1\right)},\sum \;|\;w_{1},\ldots ,w_{n}\right). \end{array}$$

Update (12) can be shown to be a weighted residual sum‐of‐squares with solution

$$\beta^{\left(l+1\right)}={\left\{\overset{n}{\underset{i=1}{\sum }}{\tilde{X}}_{i}^{T}C{\left(w_{i},\sum^{\left(l\right)}\right)}^{-1}{\tilde{X}}_{i}\right\}}^{-1}\overset{n}{\underset{i=1}{\sum }}{\tilde{X}}_{i}^{T}C{\left(w_{i},\sum^{\left(l\right)}\right)}^{-1}{\tilde{y}}_{i},$$

where ${\tilde{X}}_{i}=\nabla^{2}c(w_{i})X_{i}$ and ${\tilde{y}}_{i}=y_{i}-\nabla c(w_{i})+\nabla^{2}c(w_{i})w_{i}$. Minimizing $h_{n}$ with respect to ∑ is nontrivial owing to nonconvexity and the constraint that ∑ is positive semi‐definite. One possibility is to parameterize ∑ in a way that lends itself to unconstrained optimization (see eg, Pinheiro and Bates^33^) and use a generic solver. However, such parameterizations are inconvenient since we, as discussed in Section 2, sometimes restrict diagonal elements of ∑ to be equal to a prespecified constant for identifiability. Similarly, testing correlation of responses requires constraining some off‐diagonal elements to equal zero. Thus, we need an algorithm that allows restrictions on the elements of ∑ and ensures estimates are positive semidefinite, and can be constrained to be positive definite if desirable.

By picking an appropriate (convex) $𝕄\subseteq {\mathbb{R}}^{r}\times r$, (13) can be characterized as an optimization problem over ${\mathbb{R}}^{r}\times r$ with the constraint that ∑∈𝕄. To handle both the nonconvexity and general constraints, we propose to solve this problem using a variation of the inertial proximal algorithm proposed by Ochs et al.^34^ This is an accelerated projected gradient descent algorithm that can be used to minimize an objective function which is the sum of a nonconvex smooth function and convex nonsmooth function. In our case, $h_{n}$ (as a function of ∑) is the nonconvex smooth function and the convex nonsmooth function is the function which equals ∞ if ∑∉𝕄 and zero otherwise. This algorithm, like many popular accelerated first order algorithms, for example, FISTA,^35^ uses “inertia” in the sense that the search point is informed by the direction of change from the previous iteration, which can lead to faster convergence.

To summarize briefly, our algorithm for (13) has (t+1)th iterate $\sum^{\left(t+1\right)}={𝒫}_{𝕄}[\sum^{\left(t\right)}-\alpha \nabla_{\sum }h_{n}(\beta ,\sum^{\left(t\right)}\;|\;w_{1},\ldots ,w_{n})+\gamma \{\sum^{\left(t\right)}-\sum^{\left(t-1\right)}\}]$, where γ∈(0,1), α is determined using backtracking line search (see algorithm 4 of Ochs et al^34^), and ${𝒫}_{𝕄}$ is the projection onto 𝕄. We assume the projection is defined; it suffices, for example, that 𝕄 is nonempty, closed, and convex (corollary 5.1.19 of Megginson^36^). In our software, 𝕄 is the intersection of a set of matrices with constrained elements and the set of symmetric matrices with eigenvalues bounded below by ϵ≥0, where ϵ=0 ensures positive semidefiniteness and ϵ>0 positive definiteness. To compute projections onto 𝕄 of this form, we implement Dykstra's alternating projection algorithm.^37^ This algorithm iterates between projections onto each of the two sets whose intersection defines 𝕄. Both projections can be computed in closed form, so this algorithm tends to be very efficient. The gradient of $h_{n}$ with respect to ∑ needed for implementing the algorithm can be found in the Supplementary Material. The algorithm is terminated when the objective function values converge.

### 3.3 Updating the approximation points

We use a trust region algorithm for updating $w_{i},i=1,\ldots ,n$ (eg, chapter 4 of Nodecal and Wright^38^). Essentially, the trust region algorithm approximates the objective function locally by a quadratic and requires the computation of gradients and Hessians. The gradient is given in the Supplementary Material and the Hessian is, assuming $\sum^{-1}$ is positive definite, for i=1,…,n, $\nabla_{w_{i}}^{2}\log f_{\theta }(y_{i},w_{i})=-\nabla^{2}c(w_{i})-\sum^{-1}$. Since $\nabla^{2}c(w_{i})$ and $\sum^{-1}$ are positive definite and the latter does not depend on $w_{i}$, the objective function is strongly concave and therefore has a unique maximizer and stationary point. In practice, however, ∑ can be singular or near‐singular and the Hessian $-\sum^{-1}-\nabla^{2}c(w)$ can have a large condition number. To improve stability, we regularize by (i) adding an $L_{2}$‐penalty on $w_{i}-X_{i}\beta$ and (ii) replacing ∑ by $\underline{\sum }=\sum +\kappa I_{r}$ for some small κ>0. Then the optimization problem for updating $w_{i}$ is

$$\underset{w\in {\mathbb{R}}^{r}}{\text{arg min}}\left\{-y_{i}^{T}w+c(w)+\frac{1}{2}{\left(w_{i}-X_{i}^{T}\beta \right)}^{T}{\underline{\sum }}^{-1}(w_{i}-X_{i}\beta )+\tau \|w_{i}-X_{i}^{T}\beta {\|}^{2}\right\},$$

where τ≥0. The intuition for shrinking $w_{i}$ to $X_{i}\beta$ is that the latter is the mean of $W_{i}$ when β is the true parameter. The penalty and regularization of ∑ are only included in the update for $w_{i}$, not in the objective function for updating β and ∑. In the Supplementary Materials, we outline a procedure for obtaining starting values that can improve computing times and the quality of the resulting estimates relative to naive starting values.

## 4 APPROXIMATE LIKELIHOOD RATIO TESTING

To make inferences about the parameters we use the approximate negative log‐likelihood $h_{n}(\beta ,\sum \;|\;w_{1},\ldots ,w_{n})$, where $w_{1},\ldots ,w_{n}$ are held at the final iterates given by Algorithm 1. Focus is on testing hypotheses of the form $(\beta ,\sum )\in {ℍ}_{0}$ vs $(\beta ,\sum )\in {ℍ}_{A}$, where ${ℍ}_{0}$ and ${ℍ}_{A}$ partition the parameter set. If the null hypothesis constrains β only, we write for simplicity $\beta \in {ℍ}_{0}$, and similarly with $\sum \in {ℍ}_{0}$ if the null hypothesis constrains ∑ only. We propose the test statistic $T_{n}=h_{n}(\tilde{\beta },\tilde{\sum }\;|\;{\tilde{w}}_{1},\ldots ,{\tilde{w}}_{n})-h_{n}(\bar{\beta },\bar{\sum }\;|\;{\tilde{w}}_{1},\ldots ,{\tilde{w}}_{n})$, where $\left(\tilde{\beta },\tilde{\sum }\right)$ and the approximation points $\tilde{w}=\{{\tilde{w}}_{1},\ldots ,{\tilde{w}}_{n}\}$ are obtained by running Algorithm 1 with the restrictions implied by ${ℍ}_{0}$ and $\left(\bar{\beta },\bar{\sum })={\text{arg min}}_{(\beta ,\sum )\in {ℍ}_{0}\cup {ℍ}_{A}}h_{n}(\beta ,\sum |{\tilde{w}}_{1},\ldots ,{\tilde{w}}_{n}\right)$. That is, $\left(\tilde{\beta },\tilde{\sum }\right)$ and $\tilde{w}$ are estimates and expansion points, respectively, from fitting the null model while $\bar{\beta }$ and $\bar{\sum }$ are obtained by maximizing the working likelihood from (9) with the expansion points fixed at those obtained by fitting the null model. We fix the expansion points to ensure $\left(\tilde{\beta },\tilde{\sum }\right)$ and $\left(\bar{\beta },\bar{\sum }\right)$ are maximizers of the same working likelihood, but under different restrictions. We chose the null model's expansion points to be conservative; that is, to favor the null hypothesis model. If the working model is accurate and ${ℍ}_{0}$ contains no boundary points of the parameter set, we expect $T_{n}$ to be, under the null hypothesis, approximately chi‐square distributed with degrees of freedom equal to the number of restrictions implied by ${ℍ}_{0}$.

A main motivation for our method is inference on the covariance matrix ∑. Null models corresponding to hypotheses that constrain elements of ∑ are straightforward to fit by including those constraints in the definition of the set 𝕄 in the update for ∑. For example, to test whether the first and second element of Y are independent, fitting the null model corresponds to setting

$$𝕄=\{\sum \in {𝕊}_{+}^{r}:\sum_{12}=\sum_{21}=0\},$$

assuming there are no other restrictions. Similarly, independence of more than two responses can be tested by including more off‐diagonal restrictions in the definition of 𝕄, and equality of variances for some responses can be tested by including restrictions such as $\sum_{11}=\sum_{22}$. In principle our method could also be used to test restrictions such as $\sum_{11}=0$, corresponding to the first latent variable being constant. However, any null hypothesis which forces some eigenvalues of ∑ to be zero corresponds to testing of boundary points, and then the likelihood ratio test‐statistic has a different asymptotic distribution.^39^, ^40^, ^41^

A formal statement of the full algorithm for hypothesis testing is given in Algorithm 2. We investigate the size and power of the proposed procedure in Section 5.5.

Algorithm 2 **Hypothesis testing procedure for $(\beta ,\sum )\in {ℍ}_{0}$ vs $(\beta ,\sum )\in {ℍ}_{A}$
**

1

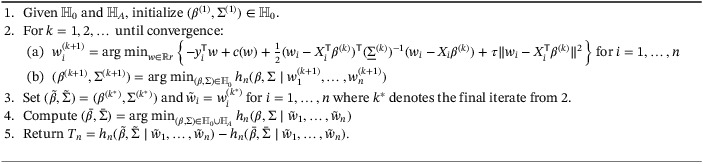

## 5 NUMERICAL EXPERIMENTS

### 5.1 Overview

We are not aware of a publicly available (or otherwise) software that fits our model outside of special cases. We therefore compare to existing methods which assume related but somewhat different models. Specifically, when investigating prediction (Section 5.2) and estimation of β (Supplementary Material) we compare to separate GLMMs for the different response types. We chose this comparison because the GLMMs have correctly specified marginal distributions in our setting. In particular, the marginal moments $𝔼(Y_{j})$ and $\mathrm{var}(Y_{j})$, j=1,…,r, are correctly specified under the GLMMs. Thus, we expect the GLMMs to give reasonable estimates of the mean functions, and expect differences in predictive performance to our method to be indicative of the usefulness of joint modeling and estimating the off‐diagonal elements of ∑. We also compare our method on prediction, estimation of β, and estimation of cor(Y) to multivariate covariance generalized linear models (MCGLMs).^29^ MCGLMs model the marginal moments of Y and, accordingly, provide estimates of the mean vector and covariance matrix of Y, but not of ∑. To further highlight the usefulness of modeling dependence, we include results for our method with ∑ constrained to be diagonal, that is, with responses assumed independent. Whether constraining ∑ to be diagonal or not, diagonal elements corresponding to Bernoulli responses are constrained to equal one when using our method.

### 5.2 Prediction comparisons

We consider r=9 response variables, three normally distributed, three Poisson, and three Bernoulli. When fitting separate GLMMs for the three‐dimensional response vectors with elements of the same type, we consider two covariance structures that common software can fit: (i) $\sum_{jj}=\sigma_{j}^{2}$ and $\sum_{jk}=0$ for j≠k or (ii) $\sum =\sigma^{2}1_{3}1_{3}^{T}$, for some $\sigma^{2}>0$ and $1_{3}={\left[1,1,1\right]}^{T}$. Option (i) assumes all responses are independent and option (ii) corresponds to using a shared random effect for observations of the same type in the same response vector. We refer to these as independent and clustered GLMMs, respectively. There are many software packages for fitting GLMMs. We pick the glmm ^23^ package to fit the independent GLMMs and fit the clustered GLMMs using lme4.^25^ Briefly, the former uses a Monte Carlo approximation of the likelihood and the latter uses adaptive Gaussian quadrature.

Predictions are formed by plugging estimates from the different methods into the expressions for $𝔼(Y_{i})=𝔼\{𝔼(Y_{i}\;|\;W_{i})\}$ in Section 2, and for simplicity we define prediction errors as the differences between those expectations and the observed responses, regardless of type. When a closed form expression is unavailable, the expectation is obtained by r one‐dimensional numerical integrations. We compare to (oracle) predictions using the true β and ∑.

We next describe how data are generated in the simulations. The responses have different predictors and β is partitioned accordingly: $\beta ={\left[\beta_{1}^{T},\ldots ,\beta_{r}^{T}\right]}^{T}$, $\beta_{j}\in {\mathbb{R}}^{\mathrm{pj}}$, and $q=\sum_{j=1}^{r}p_{j}$. We write $X_{i,j}\in {\mathbb{R}}^{\mathrm{pj}}$ for the ith observation of the predictors for the jth response. In all simulations, each $X_{i,j}$ consists of a one in the first element (an intercept) and, in the remaining $p_{j}-1$ elements, independent realizations of a U[−1,1] random variable, where $p_{j}=p_{k}$ for all j and k. For j=1,…,r, the true regression coefficient $\beta_{j}$ has first element equal to $\beta_{0j}$ and all other elements chosen as independent realizations of a U[−.5,.5]. We set $\beta_{0j}=2$ if the response is normal or (quasi‐)Poisson, and equal to zero if the response is Bernoulli. Similarly, if the response is normal, we set $\psi_{j}=.01$; otherwise, we set $\psi_{j}=1$.

We consider three different structures for ∑: for some ρ∈(0,1) we set $\sum =0.5\tilde{\sum }$ where $\tilde{\sum }$ is (i) autoregressive (${\tilde{\sum }}_{jk}=\rho^{\left|j-k\right|}$); (ii) compound symmetric (${\tilde{\sum }}_{jk}=\rho 𝕀(j\neq k)+𝕀(j=k)$); or (iii) block‐diagonal, meaning ${\tilde{\sum }}_{jk}=\rho 𝕀(j\neq k)+𝕀(j=k)$ if $(j,k)\in \left\{1,4,7\right\}\times \left\{1,4,7\right\}$, $(j,k)\in \left\{2,5,8\right\}\times \left\{2,5,8\right\}$, or $(j,k)\in \left\{3,6,9\right\}\times \left\{3,6,9\right\}$ and zero otherwise. The first through third responses are normal, the fourth through sixth Bernoulli, and seventh through ninth Poisson. Hence, each of the blocks given by the structure in (iii) includes one of each response type. These structures are used to generate the data but are not imposed when fitting models. For all the structures, the GLMMs have correctly specified diagonal elements of ∑, but incorrectly specified off‐diagonal elements in general.

For each structure of ∑, we investigate the effects of the sample size (n), the number of predictors ($p_{j}$, j=1,…,r), and the correlation parameter (ρ). We present relative squared out of sample prediction errors, defined as the ratio of a method's sum of squared prediction error to the sum of squared prediction error of the oracle prediction. Averages are based on 500 independent replications, and for each replication, out of sample predictions are on an independent test set of $10^{4}$ observations.

**FIGURE 1:**
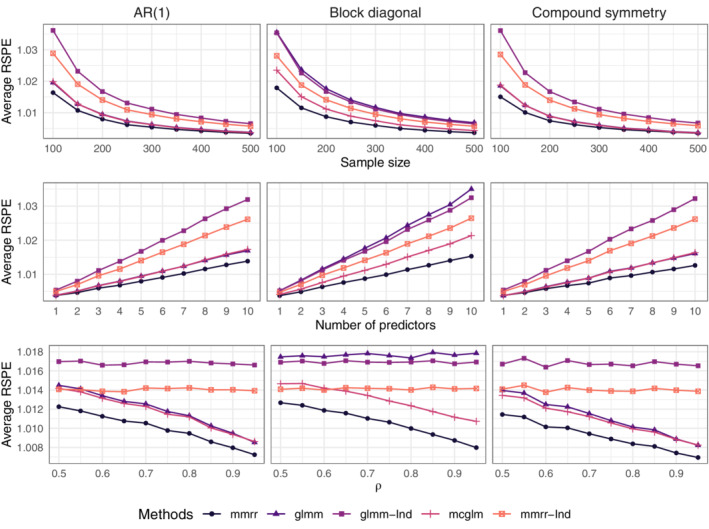
Average relative squared prediction errors. Top: ρ=0.9 and $p_{j}=5$ for j=1,…,9. Middle: n=200 and ρ=0.9. Bottom: n=200 and $p_{j}=5$ for j=1,…,9. glmm indicates clustered GLMMs, glmm‐Ind GLMMs with diagonal covariance matrix, mcglm the method of Bonat and Jørgensen,^29^ mmrr the proposed method, and mmrr‐Ind the proposed method with diagonal covariance matrix

In the top row of Figure 1, as n increases, each method's performance improves relative to oracle predictions. However, across all settings, the proposed method performs best. When the covariance structure is nonsparse (eg, autoregressive or compound symmetric), the clustered GLMMs can outperform our method with the diagonal covariance matrix and the independent GLMMs. The same relative performances are observed as p increases in the middle row; and when ρ increases in the bottom row. When ∑ is block‐diagonal, both versions of our method outperform the competitors. In the case of clustered GLMMs, this is likely due to the specified covariance structure being a poor approximation to the true covariance. For independent GLMMs, this is likely due to the fact that glmm (nor other software) can impose the identifiability condition on ∑ for the Bernoulli responses. MCGLMs perform second‐best in most settings, can perform similarly to clustered GLMMs when the true covariance structure is nonsparse, and can be outperformed by our method with diagonal covariance when dependence is weak. In summary, the results show the usefulness of joint modeling for prediction, and they illustrate the usefulness of the proposed method relative to ones based on marginal moments.

The Supplementary Material include results on mean squared errors for estimating β, for the same methods and settings used in Figure 1, and the results are qualitatively similar to those in Figure 1. The Supplementary Material also include a comparison to separate GLMs, effectively assuming independence between responses, which leads to substantially worse predictions than all methods considered here.

### 5.3 Performances for different response types

In Figure 1 averages were taken over all responses types. To see if the benefits of joint modeling are greater for some response types, it is of interest to stratify results by type. We compare the two versions of our method (diagonal vs nondiagonal ∑). Because both versions have correctly specified univariate response distributions and are fit using the same algorithm except for the constraints on ∑, these simulations investigate the usefulness of joint modeling of mixed‐type responses. Data are generated as in the previous section.

**FIGURE 2:**
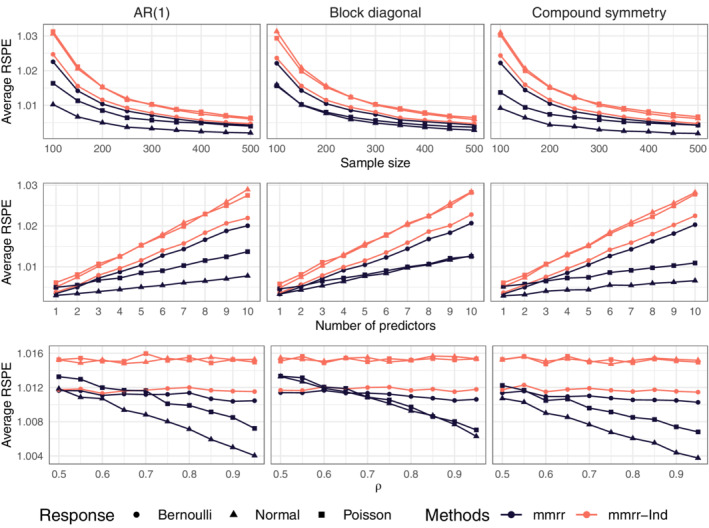
Average relative squared prediction errors. Top: ρ=0.9 and $p_{j}=5$ for j=1,…,9. Middle: n=200 and ρ=0.9. Bottom n=200 and $p_{j}=5$ for j=1,…,9. mmrr is the proposed method and mmrr‐Ind the proposed method with diagonal covariance matrix

In the first row of Figure 2, as n increases, both methods' relative mean squared prediction error approaches the oracle prediction error. However, the predictions from joint modeling outperforms those using a diagonal ∑. The differences between the two methods are smallest for Bernoulli responses. A similar result is observed in the second row: as $p_{j}$ approaches 10, both methods' relative performance degrades, although for all three response types, predictions from the joint modeling degrades more gradually. In the bottom‐most row, we display results as ρ varies. When ρ=0.5 there is a less substantial difference between the two methods. As ρ approaches 0.95, the difference between the two methods becomes greater. This result is also observed in the Bernoulli responses, but to a lesser degree than the normal and Poisson.

In Section F.2 of the Supplementary Material, we present a simulation study which focuses on modeling many Bernoulli responses and a single normal response. Those results highlight that even though the relative squared prediction error for the Bernoulli responses is only slightly improved by joint modeling, one can realize substantial prediction accuracy gains for the single normal response variable by exploiting dependence between it and the Bernoulli responses. We present similar results comparing our method and MCGLMs in the Supplementary Material.

### 5.4 Covariance estimation

To investigate the usefulness of the proposed method for estimating covariances and correlations specifically, we consider a setting without predictors. That is, we consider (2) with x=1, corresponding to an intercept only. Ideally we would compare to an established method for estimating ∑ in our setting. Since none is available (to the best of our knowledge), we compare the estimates of Ω=cov(Y) and $\text{cor}(Y)=\text{diag}{\left(\Omega \right)}^{-1/2}\Omega \text{diag}{\left(\Omega \right)}^{-1/2}$ from our method to moment‐based estimates. One is the empirical (or sample) covariance matrix $S=n^{-1}\sum_{i=1}^{n}(y_{i}-\bar{y}){\left(y_{i}-\bar{y}\right)}^{T}$, where $\bar{y}=n^{-1}\sum_{i=1}^{n}y_{i}$ is the sample mean. The corresponding empirical correlation matrix is $\text{diag}{\left(S\right)}^{-1/2}S\text{diag}{\left(S\right)}^{-1/2}$. MCGLMs also provide a moment‐based estimate of cor(Y), which we found to be indistinguishable from the empirical correlation matrix in the present setting without predictors.

We note the comparison to S, or other moment‐based estimates of cov(Y) not assuming (2), is not ideal because S cannot in general be mapped to an estimate of ∑. Formally the issue is that while Ω is injective as a function of ∑, which follows from Theorem 1, that function is not onto ${𝕊}_{+}^{r}$. Put differently, not all realizations of S give an estimate consistent with (2). Nevertheless, S is (strongly) consistent for Ω when there are no predictors since the observations are then i.i.d., so we expect reasonable estimates of Ω.

**FIGURE 3:**
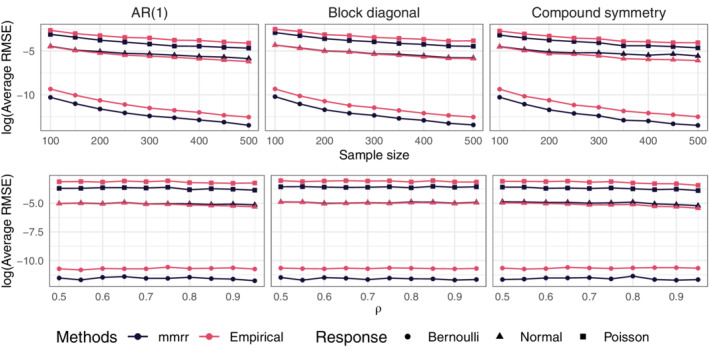
Average relative mean squared error for estimating the diagonals of cov(Y) stratified by response type. (Top row) Results with ρ=0.9. (Bottom row) Results with n=200. mmrr is the proposed method and Empirical the sample covariance

The data generating model is as in Section 5.2, but with an intercept only. Figure 3 shows average relative mean square error for the diagonal entries of Ω by type. Specifically, we report the averages of: (normal) $\sum_{j=1}^{3}{\left(\Omega_{jj}-{\hat{\Omega }}_{jj}\right)}^{2}/\sum_{j=1}^{3}\Omega_{jj}^{2}$, (Bernoulli) $\sum_{j=4}^{6}{\left(\Omega_{jj}-{\hat{\Omega }}_{jj}\right)}^{2}/\sum_{j=4}^{6}\Omega_{jj}^{2}$, and (Poisson) $\sum_{j=7}^{9}{\left(\Omega_{jj}-{\hat{\Omega }}_{jj}\right)}^{2}/\sum_{j=7}^{9}\Omega_{jj}^{2}$ where $\hat{\Omega }$ is an estimate of Ω with jth diagonal entry $\Omega_{jj}.$ In Figure 3, we see our method estimates the variances of the Poisson components more accurately than does the sample covariance, but the two perform similarly for normal and Bernoulli responses.

**FIGURE 4:**
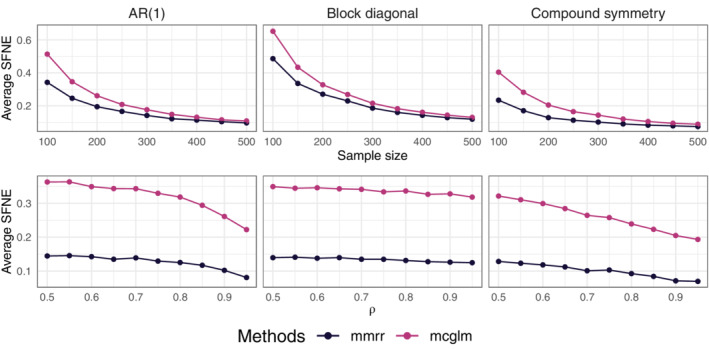
Average squared Frobenius norm error for estimating cor(Y). (Top row) Results with ρ=0.9. (Bottom row) Results with p=1 and n=200. mmrr is the proposed method and mcglm the method of Bonat and Jørgensen^29^

Figure 4 displays the average mean squared estimation error for $\text{diag}{\left(\Omega \right)}^{-1/2}\Omega \text{diag}{\left(\Omega \right)}^{-1/2}$, the correlation matrix of ${\left(Y_{1},\ldots ,Y_{9}\right)}^{⊤}$. For smaller sample sizes, the proposed method performs better than MCGLMs. For larger sample sizes the differences diminish somewhat, which is not surprising given that sample correlations are consistent. As the correlation parameter ρ varies with n=200, we see that the differences between the methods remain relatively constant, with the proposed one being better in every setting.

### 5.5 Approximate likelihood ratio testing

We examine the approximate likelihood ratio testing procedure described in Section 4. Let ${𝔻}_{++}^{r}$ be the set of r×r diagonal and positive definite matrices. We study the size and power of the proposed tests for $\sum \in {ℍ}_{0}={𝔻}_{++}^{r}$ vs $\sum \in {ℍ}_{A}={𝕊}_{++}^{r}\setminus {ℍ}_{0}$, and, assuming all responses have the same predictors as in (2), $B\in {ℍ}_{0}=\{B\in {\mathbb{R}}^{p\times r}:B_{kj}=0,\;j=1,\ldots ,r\}$ vs $B\in {ℍ}_{A}={\mathbb{R}}^{p\times r}\setminus {ℍ}_{0}$, where $B_{kj}$ denotes the kth predictor's effect on the jth response variable, that is, the (k,j)th element of B. We set k=2. Thus, the null hypothesis implies the first predictor (ignoring the intercept) has no effect on any response. Multiple testing corrections, which are often needed when using separate models for the r responses, are not needed here.

**FIGURE 5:**
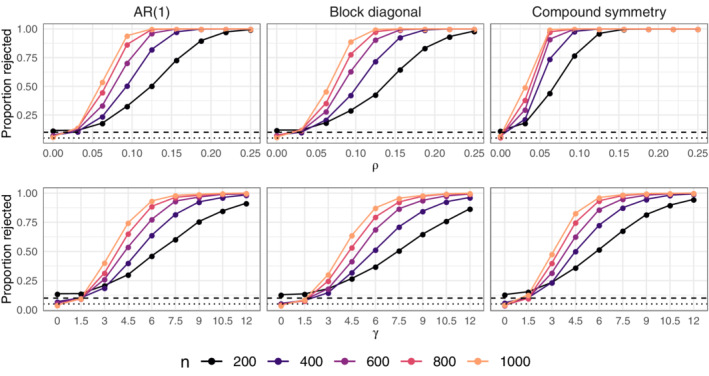
Top: Proportion of $\sum \in {ℍ}_{0}={𝔻}_{++}^{r}$ rejected at 0.05 level. Bottom: Proportion of $B\in {ℍ}_{0}=\{B\in {\mathbb{R}}^{p\times r}:B_{kj}=0,\;j=1,\ldots ,r\}$ rejected at the 0.05 level. Horizontal dashed and dotted black lines indicate 0.10 and 0.05

Data are generated as in Section 5.3 but with $X_{i,1}=X_{i,2}=\cdots =X_{i,r}$ for all i=1,…,n and $B=[\beta_{1},\ldots ,\beta_{r}]\in {\mathbb{R}}^{p\times r}$. In the first setting, n∈{200,400,…,1000} and ${\tilde{\sum }}_{jk}=\rho^{\left|j-k\right|}$, ρ∈{0.0,0.05,…,0.4}. The top row of Figure 5 displays the proportion of rejections at the 0.05 significance level. When ρ=0 (null hypothesis is true), the proportion of rejections is approximately 0.10 when n=200, below 0.075 when n≥400, and near 0.05 (the nominal level) when n=2000. As ρ increases, even with n=200, the proportion of correctly rejected null hypotheses is near one when ρ=0.4. The power depends positively on both the magnitude of ρ and the sample size.

In the second setting, we fix ρ=0.5 and study how the effect size of the $B_{kj}$ affects power. After generating B as in Section 5.3, for j=1,…,r independently, we replace $B_{kj}$ with a realization of a $U[-\gamma 10^{-2},\gamma 10^{-2}]$ where γ∈[0,12]. The second row of Figure 5 shows that when γ=0, so that $B_{kj}=0$ for all j=1,…,r, the proportion of rejections is slightly above 0.10 for n=200, but close to 0.05 (the nominal size) for all n≥400. There is also an indication that correlation between responses benefits power. For example, the power curves under compound symmetry tend to be above the corresponding ones under block‐diagonal structure.

## 6 DATA EXAMPLES

### 6.1 Fertility data

We consider a dataset collected on 333 women who were having difficulty becoming pregnant.^42^ The goal is to model four mixed‐type response variables related to the ability to conceive. The predictors are age and three variables related to antral follicles: small antral follicle count, average antral follicle count, and maximum follicle stimulating hormone level. Antral follicle counts can be measured via noninvasive ultrasound and are therefore often used to model fertility.

The response variables quantify the ability to conceive in different ways. Two are approximately normally distributed (square‐root estradiol level and log‐total gonadotropin level); and two are counts (number of egg cells and number of embryos). We modeled the latter using our model with conditional quasi‐Poisson distributions. We set $\psi_{j}=10^{-2}$ for continuous responses and $\psi_{j}=10^{-1}$ for counts. To illustrate how the proposed methods can be applied, we test the null hypothesis that ∑ is diagonal and find evidence against it (P‐value $<10^{-16}$) using the test described in Section 5.5. That is, there is evidence suggesting the four responses are not independent given the predictors. Fitting the unrestricted model using our software took less than three seconds on a laptop computer with 2.3 GHz 8‐Core Intel Core i9 processor. The hypothesis testing procedure took less than six seconds on the same machine.

The estimated correlation matrices for the four observed responses, $\hat{\text{cor}}(Y_{i}\;|\;X_{i}=\bar{X})$, and the latent variables, $\hat{\text{cor}}(W_{i}\;|\;X_{i})$, are, respectively,

$$\left(\begin{array}{rrrr} 1.00 & 0.01 & -0.08 & -0.09\\ 0.01 & 1.00 & -0.03 & -0.09\\ -0.08 & -0.03 & 1.00 & 0.69\\ -0.09 & -0.09 & 0.69 & 1.00 \end{array}\right)\;\;\;\;\;\;\;\;\;\text{and}\;\;\;\;\;\;\;\;\;\left(\begin{array}{rrrr} 1.00 & 0.02 & -0.09 & -0.10\\ 0.02 & 1.00 & -0.04 & -0.09\\ -0.09 & -0.04 & 1.00 & 0.74\\ -0.10 & -0.09 & 0.74 & 1.00 \end{array}\right),$$

where the variable ordering is square‐root estradiol level, log‐total gonadotropin level, number of egg cells, and number of embyros. The estimate of $\text{cor}(Y_{i}\;|\;X_{i})$ is here evaluated at $\bar{X}=\sum_{i=1}^{n}X_{i}/n$. The estimates indicate substantial positive correlation between the number of egg cells and number of embryos, whereas estradiol and gonadotropin levels appear weakly negatively correlated with these two variables.

We also test whether the small antra follicle count is a significant predictor of any of the responses after accounting for age, average antral follicle count, and maximum follicle stimulating hormone level. The number of small antra follicles (2‐5 mm) is correlated with the number of total antra follices (2‐10 mm), and it has been argued that only total antra follicle count are needed in practice.^43^ Fitting our model with $\sum \in {𝕊}_{++}^{4}$, we reject the null hypothesis that the four regression coefficients (one for each response) corresponding to antra follicle count is zero (P‐value =0.0052).

Finally, to illustrate how uncertainty can be quantitified using the approximate likelihood, we construct an approximate 95% confidence interval for $\sum_{43}$ by inverting the proposed approximate likelihood ratio test‐statistic numerically. This gives the confidence interval (0.17,0.24), which corresponds to correlations between 0.62 and 0.8. Confidence intervals for the other parameters could be constructed similarly.

### 6.2 Somatic mutations and gene expression in breast cancer

We now focus on jointly modeling common somatic mutations and gene expression measured on patients with breast cancer collected by The cancer genome atlas project (TCGA). A somatic mutation is an alteration in the DNA of a somatic cell. Somatic mutations are believed to play a central role in the development of cancer. Because somatic mutations modify gene expression, directly and indirectly, it is natural to model somatic mutations and gene expression jointly.

The somatic mutation variables are binary, indicating presence or absence of a somatic mutation in the region of a particular gene. We focus on the ten genes where a somatic mutation was present in more than 5% of subjects. Thus, we have r=20, coming from ten genes each with one response corresponding to gene expression and one to the presence of a somatic mutation. For gene expression, we model log‐transformed RPKM measurements as normal random variables. Each patient's age is included as a predictor.

**FIGURE 6:**
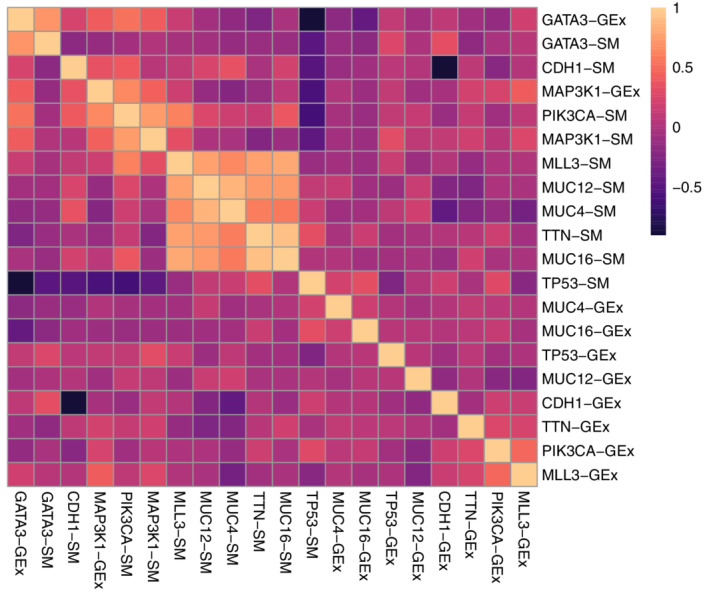
Heatmap of the estimated correlation matrix for the $W_{i}\;|\;X_{i}$ in Section 6.2. Suffix ‐SM for somatic mutations; ‐GEx for gene expression

We test the covariance matrix for block‐diagonality. Under the null hypothesis, entries of ∑ corresponding the correlations between somatic mutations and gene expression measurements are zero (ie, is no correlation between somatic mutations and gene expression). The observed statistic is $T_{n}=739$ with 100 degrees of freedom for a P‐value $<10^{-16}.$ Figure 6 displays the estimated correlation matrix for the $W_{i}|X_{i}$. We observe the latent variables corresponding to somatic mutations and gene expression in CDH1 are highly negatively correlated, whereas for GATA3, somatic mutation and gene expression latent variables have a strong positive correlation. Latent variables for many of the somatic mutations are highly correlated (eg, TTN, MLL3, MUC4, MUC12, MUC16). However latent variables corresponding to some somatic mutations, for example, those in the region of TP53, exhibit small or even negative correlations with many others (eg, GATA3, CDH1, PIK3CA). Confidence intervals could be constructed as in Example 6.1.

## 7 DISCUSSION

We have proposed a likelihood‐based method for mixed‐type multivariate response regressions. Our method gives an approximate maximum likelihood estimate of an unstructured covariance matrix characterizing the dependence between responses. This is particularly useful in settings where a dependence structure is not suggested by subject‐specific knowledge or one wants to discover such a structure using data. To address the computational challenges with estimating an unstructured covariance matrix, we have proposed a new algorithm. The algorithm handles identifiability constraints and it scales well in the dimension of the response vectors.

An advantage of likelihood‐based methods compared to ones based on marginal moments, such as for example generalized estimating equations and its extensions,^27^, ^28^, ^29^, ^44^ is the plethora of existing procedures for inference and model selection using likelihoods. For example, Wald tests and standard errors for the coefficient estimates, based on the observed Fisher information, are readily available once maximum likelihood estimates have been computed, as are information criteria. We also used the likelihood to propose a testing procedure for the covariance matrix. On the other hand, it is often computationally expensive to evaluate the likelihood in latent variable models and it can lead to complicated optimizations. We addressed that using a PQL‐like approximation and a new algorithm, and showed the resulting approximate maximum likelihood estimates are often useful. Extending our method to other likelihood‐approximations is a possibility. For example, the PQL‐approximation could be replaced by a Laplace or Monte Carlo approximation. Another potential line of future research is the asymptotic properties of PQL‐type estimators, which despite the estimators' apparent practical usefulness, are not fully understood.

Finally, we note some types of mixed‐type longitudinal data are handled by our method as‐is, while other types would require modifications or would be better analyzed using methods developed for that purpose. For example, our method can be applied when the elements of $Y_{i}\in {\mathbb{R}}^{r}$, i=1,…,n, are repeated measures of mixed‐type responses on independently sampled patients indexed by i. Modifications may be necessary if one wants to impose a particular structure on ∑, if n<r, or if there is dependence between patients.

## Supporting information


**Data S1** Supplementary Material

## Data Availability

Data sharing is not applicable to this article as no new data were created or analyzed in this study.
