# Supplementary material for: Mixed‐type multivariate response regression with covariance estimation
Source: Stat Med. 2022 Mar 24;41(15):2768–85. doi: 10.1002/sim.9383 (PMC9313904; doi:10.1002/sim.9383)
Supplement: Supplementary file 1 — Data S1 Supplementary Material [file SIM-41-2768-s001.pdf]

Supplement to "Mixed-type multivariate response regression with covariance estimation"

Karl Oskar Ekvall* Aaron J. Molstad†

karl.oskar.ekvall@ki.se amolstad@ufl.edu

Division of Biostatistics, Institute of Environmental Medicine, Karolinska Institute*

Applied Statistics Research Unit, Inst. of Stat. and Math. Methods in Econ., TU Wien*

Department of Statistics and Genetics Institute, University of Florida†

Jan 2022

A Moment calculations

We compute the moments in Example 2 that include conditionally quasi-Poisson distributed responses. We use repeatedly that the moment generating function for $\mathrm{N}(\mu, \sigma^2)$ is $M(t) = \exp(t\mu + t^2\sigma^2/2)$. First, $\mathbb{E}(Y_j) = \mathbb{E}[\mathbb{E}(Y_j \mid W_j)] = \mathbb{E}[\exp(W_j)] = \exp(X_j^\mathsf{T}\beta + \Sigma_{jj}/2)$. Similarly, for $j = 3, 4$,

$$\begin{aligned}
\mathbb{E}[Y_j^2] &= \mathbb{E}[\mathbb{E}(Y_j^2 \mid W_j)] \\
&= \mathbb{E}[\mathrm{var}(Y_j \mid W_j) + \mathbb{E}(Y_j \mid W_j)^2] \\
&= \mathbb{E}[\psi_j \exp(W_j)] + \mathbb{E}[\exp(2W_j)] \\
&= \psi_j \exp(X_j^\mathsf{T}\beta + \Sigma_{jj}/2) + \exp(2X_j^\mathsf{T}\beta + 2\Sigma_{jj}),
\end{aligned}$$

where we used $2W_j \sim \mathrm{N}(2X_j^\mathsf{T}\beta, 4\Sigma_{jj})$. It follows that, for $j = 3, 4$,

$$\begin{aligned}
\mathrm{var}(Y_j) &= \mathbb{E}(Y_j^2) - \mathbb{E}(Y_j)^2 \\
&= \psi_j \exp(X_j^\mathsf{T}\beta + \Sigma_{jj}/2) + \exp(2X_j^\mathsf{T}\beta + 2\Sigma_{jj}) - \exp(2X_j^\mathsf{T}\beta + \Sigma_{jj}) \\
&= \exp(2X_j^\mathsf{T}\beta + \Sigma_{jj}) \left[\psi_j \exp(-X_j^\mathsf{T}\beta - \Sigma_{jj}/2) + \exp(\Sigma_{jj}) - 1\right].
\end{aligned}$$

To get the covariance $\mathrm{cov}(Y_j, Y_k)$ for $j = 1, 2$ and $k = 3, 4$, observe that since Y_j and Y_k are uncorrelated given W,

$$\begin{aligned}
\mathrm{cov}(Y_j, Y_k) &= \mathrm{cov}[\mathbb{E}(Y_j \mid W), \mathbb{E}(Y_k \mid W)] \\
&= \mathrm{cov}[W_j, \exp(W_k)] \\
&= \mathbb{E}[W_j \exp(W_k)] - X_j^\mathsf{T}\beta \exp(X_k^\mathsf{T}\beta + \Sigma_k/2),
\end{aligned}$$

and

$$\mathbb{E}[W_j \exp(W_k)] = \mathbb{E}\left[\frac{\partial}{\partial t_j} \exp(t_j W_j + t_k W_k) \mid_{t_j=0,t_1=1}\right].$$

Now, for (t_j, t_k) in a neighborhood of $(0, 1)$,

$$\left|\frac{\partial}{\partial t_j} \exp(t_j W_j + t_k W_k)\right| = |W_j \exp(t_j W_j + t_k W_k)| \leq \exp(|W_j|) \exp(|W_j| + |W_k|),$$

which has finite expectation since W_j and W_k are jointly normal. Thus, we can move the derivative outside the expectation to get

$$\begin{aligned}
\mathbb{E}[W_j \exp(W_k)] &= \frac{\partial}{\partial t_j}\mathbb{E}\left[\exp(t_j W_j + t_k W_k)\right] \mid_{t_j=0,t_1=1} \\
&= \frac{\partial}{\partial t_j} \exp\left(t_j X_j^\intercal \beta + t_k X_k^\intercal \beta + t_j^2 \Sigma_{jj}^2/2 + t_j t_k \Sigma_{jk} + t_k^2 \Sigma_{kk}/2\right) \mid_{t_j=0,t_1=1} \\
&= (X_j^\intercal \beta + \Sigma_{jk}) \exp\left(X_k^\intercal \beta + \Sigma_{kk}/2\right)
\end{aligned}$$

where in the second equality we used the moment generating function for

$$t_j W_j + t_k W_k \sim \mathrm{N}\left(t_j X_j^\intercal \beta + t_k X_k^\intercal \beta, t_j^2 \Sigma_{jj}^2 + 2 t_j t_k \Sigma_{jk} + t_k^2 \Sigma_{kk}\right).$$

Putting things together, we have

$$\begin{aligned}
\operatorname{cov}(Y_j, Y_k) &= (X_j^\intercal \beta + \Sigma_{jk}) \exp\left(X_k^\intercal \beta + \Sigma_{kk}/2\right) - X_j^\intercal \beta \exp\left(X_k^\intercal \beta + \Sigma_{kk}/2\right) \\
&= \Sigma_{j,k} \exp\left(X_k^\intercal \beta + \Sigma_{kk}/2\right).
\end{aligned}$$

Lastly, we compute

$$\begin{aligned}
\operatorname{cov}(Y_3, Y_4) &= \operatorname{cov}[\exp(W_3), \exp(W_4)] \\
&= \mathbb{E}[\exp(W_3)\exp(W_4)] - \mathbb{E}[\exp(W_3)]\mathbb{E}[\exp(W_4)] \\
&= \mathbb{E}[\exp(W_3 + W_4)] - \exp(X_3^\intercal \beta + \Sigma_{33}/2 + X_4^\intercal \beta + \Sigma_{44}/2) \\
&= \exp(X_3^\intercal \beta + \Sigma_{33}/2 + X_4^\intercal \beta + \Sigma_{44}/2) \left[\exp(\Sigma_{34}) - 1\right],
\end{aligned}$$

where, as before, the last step used the moment generating function for the normal variable $W_3 + W_4$.

B Proofs

Lemma B.1. *Let $W \sim \mathrm{N}(\mu, \sigma^2)$ and Φ denote the standard normal cumulative distribution function, then*

$$\mathbb{E}[\Phi(W)] = \Phi(\mu/\sqrt{1 + \sigma^2}))$$

Proof. This is well known and is, for example, essentially Equation 10 in McCulloch (2008). □

Let $\phi_\sigma(u,v)$ be the bivariate normal density mean zero, unit variances, and covariance σ.

Lemma B.2. *The function h defined by*

$$h(\sigma,c_1,c_2)=\frac{\partial}{\partial\sigma}\iint I(u>c_1)I(v>c_2)\phi_\sigma(u,v)\mathrm{d}u\mathrm{d}v$$

is strictly positive and continuous on $(-1,1)\times\mathbb{R}\times\mathbb{R}$.

Proof. We first prove h is strictly positive. Let U and V denote random variables with density $\phi_\sigma(u,v)$. By using that $U\mid V\sim\mathrm{N}(\sigma V,1-\sigma^2)$ and letting Φ denote the standard normal cumulative distribution function,

$$\begin{aligned}\mathbb{E}[I(U>c_1)I(V>c_2)]&=\mathbb{E}\left\{I(V>c_2)\mathbb{E}\left[I(U>c_1)\mid V\right]\right\}\\&=\mathbb{E}\left[I(V>c_2)\mathsf{P}(U>c_1\mid V)\right]\\&=\mathbb{E}\left\{I(V>c_2)\left[1-\Phi\left(\frac{c_1-\sigma V}{\sqrt{1-\sigma^2}}\right)\right]\right\}\\&=\mathsf{P}(V>c_2)-\mathbb{E}\left[I(V>c_2)\Phi\left(\frac{c_1-\sigma V}{\sqrt{1-\sigma^2}}\right)\right].\end{aligned}$$

Denote the expectation in the last line by $J_1(\sigma,c_1,c_2)$; we want to show that $\partial J_1(\sigma,c_1,c_2)/\partial\sigma<0$. Differentiating under the integral we find

$$\int_{c_2}^{\infty}\phi\left(\frac{c_1-\sigma v}{\sqrt{1-\sigma^2}}\right)\frac{c_1\sigma-v}{(1-\sigma^2)^{3/2}}\phi(v)\mathrm{d}v,$$

where ϕ is the standard normal probability density function. Differentiating under the integral is permissible because $\phi(\cdot)$, $1/(1-\sigma^2)^{3/2}$, and σ are all bounded on small enough neighborhoods of any $\sigma\in(-1,1)$. Now, if $c_2\geq\sigma c_1$ the integrand is negative on the set of integration we are done. Suppose thus $c_2<\sigma c_1$, and note

$$\begin{aligned}\mathbb{E}\left[\Phi\left(\frac{c_1-\sigma V}{\sqrt{1-\sigma^2}}\right)\right]&=\mathbb{E}\left[I(V>c_2)\Phi\left(\frac{c_1-\sigma V}{\sqrt{1-\sigma^2}}\right)\right]+\mathbb{E}\left[I(V\leq c_2)\Phi\left(\frac{c_1-\sigma V}{\sqrt{1-\sigma^2}}\right)\right]\\&=J_1(\sigma,c_1,c_2)+J_2(\sigma,c_1,c_2),\end{aligned}$$

where J_2 is defined by the last equality. Lemma B.1 says the left hand side is

$$\mathbb{E}\left[\Phi\left(\frac{c_1/\sqrt{1-\sigma^2}}{\sqrt{1+\sigma^2/(1-\sigma^2)}}\right)\right]=\Phi(c_1).$$

Thus, differentiating both sides with respect to σ gives

$$0=\frac{\partial}{\partial\sigma}J_1(\sigma,c_1,c_2)+\frac{\partial}{\partial\sigma}J_2(\sigma,c_1,c_2),$$

so it suffices to show the last term is positive. But by argument similar to when differentiating J_1,

$$\frac{\partial}{\partial\sigma}J_2(\sigma, c_1, c_2) = \int_{-\infty}^{c_2} \phi\left(\frac{c_1 - \sigma v}{\sqrt{1-\sigma^2}}\right) \frac{c_1\sigma - v}{(1-\sigma^2)^{3/2}}\phi(v)\mathrm{d}v,$$

which is positive since the integrand is positive on the set of integration. Finally, that $h(\sigma, c_1, c_2)$ is continuous follows from the dominated convergence theorem since the integrand is bounded on small enough neighborhoods around any interior point of $(-1, 1) \times \mathbb{R} \times \mathbb{R}$. $\square$

Lemma B.3. *If $f, g : \mathbb{R} \to \mathbb{R}$ are increasing and non-constant, and*

$$\iint |f(u)||g(v)|\phi_\sigma(u, v)\mathrm{d}u\mathrm{d}v < \infty$$

for all $\sigma \in (-1, 1)$, then $s : (-1, 1) \to \mathbb{R}$ defined by

$$s(\sigma) = \iint f(u)g(v)\phi_\sigma(u, v)\mathrm{d}u\mathrm{d}v$$

is strictly increasing.

Proof. First observe that since the marginal densities do not depend on u and v, we may replace f and g by $f - f(0)$ and $g - g(0)$; that is, we assume without loss of generality that $f(0) = g(0) = 0$.

For every $n = 1, 2, \ldots$ and $i = 0, \ldots, n2^{n+1} = m_n$, let $a_{ni} = -n + i/2^n$. Then for every n,

$$-n = a_{n0} < \cdots < a_{m_n/2} = 0 < \cdots < a_{m_n} = n.$$

and the distance between consecutive a_{ni} is $1/2^n$. Define

$$f_n^-(u) = f(a_{n0}) + \sum_{i=1}^{m_n/2}[f(a_{ni}) - f(a_{n(i-1)})]I(u \geq a_{n(i-1)}),$$

$$f_n^+(u) = \sum_{i=m_n/2+1}^{m_n}[f(a_{ni}) - f(a_{n(i-1)})]I(u \geq a_{ni}),$$

and

$$f_n = f_n^- + f_n^+.$$

Note that if $u \geq -1/2^n$, then $f_n^-(u) = f(a_{m_n/2}) = 0$, and if $u < 1/2^n$, then $f_n^+(u) = 0$. Thus, $f_n(0) = 0$ for every n and at most one of f_n^- and f_n^+ are non-zero for the same u. If $u < 0$, then $f_n(u) = f_n^-(u) = f(a_{nj})$ where a_{nj}, $j = j(n)$, is the smallest a_{ni} greater than u. Since f is increasing, $0 \geq f(a_{nj}) \geq f(u)$ and if u is a point of continuity of f, $f(a_{nj}) \downarrow f(u)$. Because f is increasing, it has at most countably many points of discontinuity and hence, for Lebesgue-almost every $u < 0$, $f_n(u) \downarrow f(u)$. A similar argument shows $0 \leq f_n(u) \uparrow f(u)$ for

Lebesgue-almost every $u > 0$. Thus, $|f_n| \leq |f|$ and $f_n \to f$ for Lebesgue-almost every u. For simplicity, we write

$$f_n(u) = f(-n) + \sum_{i=1}^{m_n} d^f_{ni} I(u \geq c_{ni}),$$

where $d_{ni} = f(a_{ni}) - f(a_{n(i-1)})$ and $c_{ni} = a_{n(i-1)}$ for $i = 1, \ldots, m_{n/2}$ and $c_{ni} = a_{ni}$ for $i = m_n/2 + 1, \ldots, m_n$. Note that the d^f_{ni} are non-negative since f is increasing.

Define h_n as f_n but with g playing the role of f, so that

$$h_n(v) = g(-n) + \sum_{i=1}^{m_n} d^g_{ni} I(v \geq c_{ni}).$$

Now with $s_n(\sigma) = \iint f_n(u) h_n(v) \phi_\sigma(u, v) \mathrm{d}u\mathrm{d}v$ and $\sigma_1 > \sigma_2$,

$$s_n(\sigma_1) - s_n(\sigma_2) = \sum_{i=1}^{m_n} \sum_{j=1}^{m_n} d^f_{ni} d^g_{nj} \iint I(u \geq c_{ni}) I(v \geq c_{nj}) [\phi_{\sigma_1}(u, v) - \phi_{\sigma_2}(u, v)] \mathrm{d}u\mathrm{d}v$$

Lemma B.2 implies all summands are non-negative; to show some summands are strictly positive, note that since f is non-constant, we can find $-\infty < l_f < u_f < -\infty$ such that

$$\lim_{u \uparrow l_f} f(u) \leq \lim_{u \downarrow l_f} f(u) < \lim_{u \uparrow u_f} f(u) \leq \lim_{u \downarrow u_f} f(u).$$

Similarly, we can find $l_g < u_g$ with the same property for g. Now, since all summands are non-negative, the sum is made no smaller by only retaining some summands. Specifically, let us retain only those i for which both a_{ni} and $a_{n(i-1)}$ are in $[l_f, u_f]$ and those j for which both a_{nj} and $a_{n(j-1)}$ are in $[l_g, u_g]$.

For such summands, by the mean value theorem, applicable owing to Lemma B.2,

$$\iint I(u \geq c_{ni}) I(v \geq c_{nj}) [\phi_{\sigma_1}(u, v) - \phi_{\sigma_2}(u, v)] \mathrm{d}u\mathrm{d}v = h(\tilde{\sigma}, c_{ni}, c_{nj})$$

for some $\tilde{\sigma}$ between σ_1 and σ_2. By Lemma B.2, h is continuous and strictly positive on the compact $[\sigma_1, \sigma_2] \times [l_f, u_f] \times [l_g, u_g]$, and hence attains a strictly positive infimum there, say $\epsilon > 0$. Thus,

$$s_n(\sigma_1) - s_n(\sigma_2) \geq \epsilon \sum_i \sum_j d^f_{ni} d^g_{nj} = \epsilon \left[\sum_i d^f_{ni} \right] \left[\sum_j d^g_{nj} \right],$$

where the sums are over the retained indexes, which are consecutive. Consider the first sum: it is the sum of jumps of f_n in $[l_f, u_f]$, and hence it tends to $\lim_{u \uparrow u_f} f(u) - \lim_{u \downarrow l_f} f(u) > 0$. Similarly, the second sum tends to $\lim_{v \uparrow u_g} g(v) - \lim_{v \downarrow l_g} g(v) > 0$. Thus, we can find a $c > 0$ such that for all n large enough, $s_n(\sigma_1) - s_n(\sigma_2) \geq c$, and the proof is completed by sending n to infinity and applying the dominated convergence theorem – the dominating function can be $|fg|\phi_{\sigma_i} \geq |f_n||h_n|\phi_{\sigma_i}$, $i = 1, 2$. □

Proof of Lemma 1. By a change of variables, the fist integral is

$$\int g(\mu_1 + \sigma_1 u)\phi(u)\mathrm{d}u$$

where ϕ is the standard normal density. For $\mu_1 > \mu_1'$,

$$\int g(\mu_1 + \sigma_1 u)\phi(u)\mathrm{d}u - \int g(\mu_1' + \sigma_1 u)\phi(u)\mathrm{d}u = \int [g(\mu_1 + \sigma_1 u) - g(\mu_1' + \sigma_1 u)]\phi(u)\mathrm{d}u \\ \geq 0$$

since the integrand is non-negative due to g being increasing. Moreover, equality holds if and only if $g(\mu_1 + \sigma_1 u) = g(\mu_1' + \sigma_1 u)$ for Lebesgue-almost every u. But since g is increasing and non-constant, we can find a point s such that g is strictly greater on (s, ∞) than on $(-\infty, s)$. Thus, for all u such that $\mu_1' + \sigma_1 u < s < \mu_1 + \sigma_1 u$, which is a set of positive Lebesgue measure since $\mu_1 > \mu_1'$, it holds that $g(\mu_1 + \sigma_1 u) > g(\mu_1' + \sigma_1 u)$, and this proves the first claim.

To prove the second claim, make another change of variables to get that the integral is

$$\int g(\mu_1 + \sigma_1 u_1)h(\mu_2 + \sigma_2 u_2)\phi_C(u)\,\mathrm{d}u,$$

where ϕ_C is the bivariate normal density with the covariance matrix C that has ones on the diagonal and $\rho = \sigma/(\sigma_1\sigma_2)$ on the off-diagonal; that is, C is the correlation matrix corresponding to Σ. Since $u_1 \mapsto g(\mu_1 + \sigma_1 u_1)$ and $u_2 \mapsto h(\mu_2 + \sigma_2 u_2)$ are increasing and non-constant because g and h are, Lemma B.3 says the integral in the last display is strictly increasing in ρ, and from this the claim follows since σ_1 and σ_2 are strictly positive. □

Lemma B.4. *Suppose $Y \in \mathbb{R}^r$ has density*

$$f_\theta(y) = |\Sigma|^{-1/2} \int_{\mathbb{R}^r} \exp\left[\sum_{j=1}^r \psi_j^{-1}\{y_j w_j - c_j(w_j)\} - (w - \mu)^\mathsf{T}\Sigma^{-1}(w - \mu)/2\right] \mathrm{d}w$$

where $\theta = (\mu, \Sigma) \in \mathbb{R}^r \times \mathbb{S}^r_+$, $\psi \in (0, \infty)^r$, and the c_j are the cumulant functions for some, possibly different, one-parameter exponential families. Let $A_j \subseteq \mathbb{R}$ denote the set of possible μ_j and $B_j \subseteq [0, \infty)$ the set of possible Σ_{jj}, $j = 1, \ldots r$. Define the functions $v_j : A_j \times B_j \to \mathbb{R} \times (0, \infty)$ by

$$v_j(\mu_j, \Sigma_{jj}) = [\mathbb{E}_\theta(Y_j), \mathrm{var}_\theta(Y_j)]^\mathsf{T}.$$

If the v_j are injective, then the parameter θ is identifiable; that is, $f_\theta(y) = f_{\theta'}(y)$ for almost every y implies $\theta = \theta'$.

Proof. Pick arbitrary θ and θ' and suppose $f_\theta = f_{\theta'}$ almost everywhere. Then

$$\mathbb{E}_\theta(Y) = \int y f_\theta(y)\mathrm{d}y = \int y f_{\theta'}(y)\mathrm{d}y = \mathbb{E}_{\theta'}(Y),$$

where $\mathrm{d}y$ means integration with respect to the dominating measure for $f_\theta(y)$. Similarly, for $j = 1, \dots, r$,

$$\mathrm{var}_\theta(Y_j) = \int \left(y_j - \int y_j f_\theta(y)\mathrm{d}y\right)^2 f_\theta(y)\mathrm{d}y = \int \left(y_j - \int y_j f_{\theta'}(y)\mathrm{d}y\right)^2 f_{\theta'}(y)\mathrm{d}y = \mathrm{var}_{\theta'}(Y_j).$$

Thus, because the v_j are injective, $\mu_j = \mu_j'$ and $\Sigma_{jj} = \Sigma_{jj}'$ for $j = 1, \dots, r$.

It remains to show $\Sigma_{ij} = \Sigma_{ij}'$ for all $i \neq j$. To that end, first note $\mathbb{E}_\theta(Y_i Y_j) = \mathbb{E}_\theta\{\mathbb{E}(Y_i Y_j \mid W)\} = \mathbb{E}_\theta\{\mathbb{E}(Y_i \mid W_i)\mathbb{E}(Y_j \mid W_j)\} = \mathbb{E}_\theta\{c_i'(W_i)c_j'(W_j)\}$ by conditional independence. Moreover, by properties of cumulant functions, $c_j''(W_j) = \mathrm{var}(Y_j \mid W_j)/\psi_j > 0$, so c_j' is strictly increasing, and similarly for c_i. Thus, by Lemma 1 in the main text,

$$\Sigma_{ij} \mapsto \mathbb{E}_\theta(Y_i Y_j)$$

is strictly increasing. Thus, because $\mathbb{E}_\theta(Y_i)$ and $\mathbb{E}_\theta(Y_j)$ do not depend on Σ_{ij}, the map $\Sigma_{ij} \mapsto \mathrm{cov}_\theta(Y_i Y_j)$ is also strictly increasing, and hence injective. Thus, since

$$\mathrm{cov}_\theta(Y_i Y_j) = \mathrm{cov}_{\theta'}(Y_i Y_j)$$

by the assumption that $f_\theta = f_{\theta'}$ almost everywhere, it must be that $\Sigma_{ij} = \Sigma_{ij}'$ and this completes the proof.

□

Proof of Theorem 1. We first show that distinct parameters give distinct first and second moments of the elements of $\mathcal{Y}$. To this end, recall from Example 2 that $\mathbb{E}(Y_{i,j}) = X_{i,j}^\mathsf{T}\beta$ and $\mathrm{var}(Y_{i,j}) = \psi_j + \Sigma_{jj}$ if $Y_{i,j}$ is normal; and if it is conditionally Poisson, then $\mathbb{E}(Y_{i,j}) = \exp(X_{i,j}^\mathsf{T}\beta + \Sigma_{jj}/2)$ and

$$\begin{aligned}
\mathbb{E}(Y_{i,j}^2) &= \mathbb{E}[\mathbb{E}(Y_{i,j}^2 \mid W_i)] \\
&= \mathbb{E}[\mathrm{var}(Y_{i,j} \mid W_i) + \mathbb{E}(Y_{i,j} \mid W_i)^2] \\
&= \mathbb{E}[\exp(W_i)] + \mathbb{E}[\exp(2W_i)] \\
&= \exp(X_{i,j}^\mathsf{T}\beta + \Sigma_{jj}/2) + \exp(2X_{i,j}^\mathsf{T}\beta + 2\Sigma_{jj}).
\end{aligned}$$

Recall also from Example 3 that, owing to Lemma 2.1, $\mathbb{E}(Y_{i,j})$ is strictly increasing in $X_{i,j}^\mathsf{T}\beta$. Thus, the first and second moments of the elements of $\mathcal{Y}$ corresponding to pairs (β, Σ) and (β_*, Σ_*) are the same only if

$$X_{i,j}^\mathsf{T}\beta = X_{i,j}^\mathsf{T}\beta_* \quad \text{and} \quad \psi_j + \Sigma_{jj} = \psi_j + \Sigma_{*jj}$$

for every i and j corresponding to normal responses;

$$\exp(X_{i,j}^\mathsf{T}\beta + \Sigma_{jj}/2) = \exp(X_{i,j}^\mathsf{T}\beta_* + \Sigma_{*jj}/2) \quad \text{and} \quad \exp(2X_{i,j}^\mathsf{T}\beta + 2\Sigma_{jj}) = \exp(2X_{i,j}^\mathsf{T}\beta_* + 2\Sigma_{*jj})$$

for every i and j corresponding to conditionally Poisson responses; and $X_{i,j}^\mathsf{T}\beta = X_{i,j}^\mathsf{T}\beta_*$ for every i and j corresponding to Bernoulli responses. Since the exponential function is invertible, if $\mathcal{X} = [X_1^\mathsf{T}, \dots, X_n^\mathsf{T}]^\mathsf{T} \in \mathbb{R}^{rn \times p}$ has full column rank, this can happen only if $\beta = \beta_*$ and $\Sigma_{jj} = \Sigma_{*jj}$ for every j. Finally, the off-diagonal elements of Σ are identifiable by Lemma 2.1 since the link functions are strictly increasing. □

C Comparison to existing software

C.1 GLMM software

We illustrate using an example. Suppose there are r conditionally Poisson-distributed responses, each with its own intercept. Specifically, for $j = 1, \ldots, r$ and independently for $i = 1, \ldots, n$,

$$Y_{i,j} \mid W_i \overset{indep.}{\sim} \mathrm{Poi}(W_{i,j}), \quad W_i \sim \mathrm{N}(\beta, \Sigma), \quad (\beta, \Sigma) \in \mathbb{R}^q \times \mathbb{S}^r_{++}.$$

This model is equivalent to a generalized linear mixed model for $[Y_{1,1}, Y_{1,2}, \ldots, Y_{n,r}]^\mathsf{T} \in \mathbb{R}^{rn}$, the vector of all responses, with linear predictor

$$\eta = (1_n \otimes I_r)\beta + U,$$

where the random effects vector $U \sim \mathrm{N}(0, I_n \otimes \Sigma)$. Even with these simplifications of the model, it is not clear that common software can fit it: the Kronecker structure is supported by neither the `GLIMMIX` procedure in SAS (Schabenberger, 2005) nor any of the R functions `glmer` from the package `lme4` (Bates et al., 2015), `glmmPQL` from the package `MASS` (Venables and Ripley, 2002), `glmmTMB` from the package with the same name (Brooks et al., 2017), or `glmm` from the package with the same name (Knudson et al., 2021). Some of the packages can fit this model if Σ is constrained to be diagonal since that corresponds to including a separate random effect for each of the observed rn responses and then constraining some of the variances of those random effects to be equal. However, a diagonal Σ is equivalent to assuming all responses are independent, and hence is typically not an interesting alternative. An arguably more reasonable alternative for these data, which all of the mentioned software packages support, is to treat $Y_{i,1}, \ldots, Y_{i,r}$ as observations from the same cluster and model within-cluster dependence by including a shared random effect. That is, by considering the linear predictor

$$\eta = (1_n \otimes I_r)\beta + (I_n \otimes 1_r)U,$$

where $U \sim \mathrm{N}(0, \sigma^2 I_n)$. This implies the covariance

$$\mathrm{cov}(\eta) = I_n \otimes \sigma^2 1_r 1_r^\mathsf{T},$$

which is equivalent to taking $\Sigma = \sigma^2 1_r 1_r^\mathsf{T}$ in our model. We expect that if this structure is correct, then our method should give coefficient estimates similar to those of `glmmPQL`.

D A quasi-Poisson distribution

We say a response Y_j has conditional quasi-Poisson moments if $\mathbb{E}(Y_j \mid W) = \exp(W_j)$ and $\mathrm{var}(Y_j \mid W) = \psi_j \exp(W_j)$ for $\psi_j > 0$. To generate such responses, notice that if $\tilde{Y}_j \mid W$ is Poisson with parameter $\psi_j^{-1} \exp(W_j)$, then $Y_j = \psi_j \tilde{Y}_j$ satisfies $\mathbb{E}(Y_j \mid W) = \exp(W_j)$ and

$$\mathrm{var}(Y_j \mid W) = \mathrm{var}(\psi_j \tilde{Y}_j \mid W) = \psi_j^2 \psi_j^{-1} \exp(W_j) = \psi_j \exp(W_j),$$

as desired. That is, conditionally quasi-Poisson responses can be generated by scaling Poisson responses. The quasi-Poisson responses will in general not be integer-valued.

E Computing details

E.1 Algorithm 1 details

The gradient required for implementing the accelerated projected gradient descent algorithm can be derived as follows. Letting $r_i = \tilde{y}_i - \tilde{X}_i\beta$ and $D_i = \nabla^2 c(w_i)$, we can write

$$h_n(\beta, \Sigma \mid w_1, \dots, w_n) = \sum_{i=1}^{n} \left[\log\det\{D_i\Sigma D_i + D_i \operatorname{diag}(\psi)\} + r_i^\mathsf{T}\{D_i\Sigma D_i + D_i \operatorname{diag}(\psi)\}^{-1} r_i\right].$$

Letting $C_i(\Sigma) = \{D_i\Sigma D_i + D_i \operatorname{diag}(\psi)\}^{-1}$, for $i = 1, \dots, n$, routine calculations give

$$\nabla_\Sigma h_n(\beta, \Sigma; w_1, \dots, w_n) = \sum_{i=1}^{n} D_i \left\{C_i(\Sigma) - C_i(\Sigma) r_i r_i^\mathsf{T} C_i(\Sigma)\right\} D_i.$$

The gradient for the update of the w_i are, for $i = 1, \dots, n$, assuming Σ^{-1} exists,

$$\nabla_{w_i} \log f(w_i, y_i; \beta, \Sigma) = y_i - \nabla c(w_i) - \Sigma^{-1}(w_i - X_i^\mathsf{T}\beta).$$

Initializing values can affect the final estimates of (β, Σ). For this reason, we propose a two-step initialization approach which we find leads to good initial values. In the first step, we run Algorithm 1 after initializing $w_i = 0$, $\beta = 0$, and $\Sigma = I_r$ under the restriction that Σ is diagonal. Once this algorithm has converged, in the second step, we run Algorithm 1 again by initializing (β, Σ) and the w_i at their final iterates from the first step. However, we drop the constraint that Σ is diagonal, and allow Σ to be unrestricted (i.e., Σ need not belong to $\mathbb{M}$). We also replace step 3(b) – (c) by a trust region algorithm which often converges quickly but does not guarantee positive semi-definiteness. Once this algorithm has converged, we use the final iterates of (β, Σ) and the w_i as our initial values for Algorithm 1 under the restriction that $\Sigma \in \mathbb{M}$. In terms of computing time, we found this approach is often faster than running Algorithm 1 directly; and tends to lead to better estimates of (β, Σ). If r is relatively large, the trust region update of Σ used to get initial values can be slow since it requires repeatedly computing a Hessian of dimension $r(r + 1)/2 \times r(r + 1)/2$; the second initialization step can then be skipped.

Table A shows the times to fit our model and the models assumed by `glmm` and `glmer` in Section 5. We see that in general, our algorithm requires more time to compute than does `glmer`, both of which are significantly faster than `glmm`.

F Additional simulation results

F.1 Additional prediction results

In this subsection we provide simulation results analogous to those in Figure 2 of the main manuscript, but comparing `mmrr` and `mcglm`. These results can be found in Figure A. Note

Covariance	Model	Sample size 100	150	200	250	300	350	400	450	500
AR(1)	`mmrr`	98.2	79.1	112.1	138.6	163.8	92.6	182.2	166.5	186.1
	`glmm`	76.0	88.9	206.6	412.9	617.0	442.1	1193.7	1623.0	2213.8
	`glmer`	14.5	16.4	24.5	31.5	42.4	32.0	56.0	61.1	68.7
BD	`mmrr`	24.0	29.1	68.0	67.0	72.2	39.3	64.8	57.8	80.6
	`glmm`	45.0	81.3	267.6	414.9	612.7	368.8	904.8	778.2	1801.8
	`glmer`	6.3	9.6	20.3	24.6	31.4	22.9	37.7	30.8	52.8
CS	`mmrr`	88.7	130.4	121.2	160.9	165.2	158.4	186.7	84.9	87.6
	`glmm`	62.7	129.3	183.4	356.8	518.5	712.2	1123.6	666.4	850.2
	`glmer`	12.4	22.2	25.2	35.9	40.4	44.2	57.6	34.5	40.3

Table A: Median computing times (in seconds) for our method with off-diagonals of Σ unconstrained (`mmrr`), independent generalized linear mixed models fit using `glmm`, and clustered generalized linear models fit using `glmer` under the settings considered in the top row of Figure 1 in the main article. AR(1), BD, and CS correspond to autoregressive, block diagonal, and compound symmetric covariance structures, respectively.

`mmrr` and `mcglm` perform similarly on normal responses, but that `mmrr` outperforms `mcglm` substantially on both Poisson and to a greater extent, Bernoulli responses. Interesingly, under the block diagonal covariance structure, `mmrr` outperforms `mcglm` on even normal responses to a notable degree.

F.2 Estimation of regression coefficients and covariance parameters

In this subsection we report additional simulation study results to accompany those in Section 5 of the main manuscript.

In Figure B, we present results for the competitors considered in Figure 1 of the main manuscript in terms of mean squared estimation error of β. These results largely agree with those when using relative mean squared prediction error as a performance metric: `mmrr` tends to outperform all competitors with `mmrr-Ind` and `glmm-Ind` performing similarly. In Figure C, we aggregate results by response type and see that components of β corresponding to Bernoulli responses are estimated worst. Note that these results are presented on the log-scale: were they left on their original scale, differences between `mmrr` and `mmrr-Ind` would appear much more substantial for Bernoulli responses (with `mmrr` significantly outperforming `mmrr-Ind`) and much less substantial for Poisson and Normal.

We now turn our attention to the estimation of Σ. We compare `mmrr-Ind` and `mmrr` in terms of their mean squared error for the diagonal entries of Σ. Since both methods constrain the diagonal entries of Σ to be equal to one for Bernoulli responses, we only present results averaged over all normal responses and Poisson responses, separately. In Figure D, we see that `mmrr` and `mmrr-Ind` differ primarily in their estimation of the Poisson variance. In every case, it appears that `mmrr` outperforms `mmrr-Ind` in terms of variance estimation for the Poisson

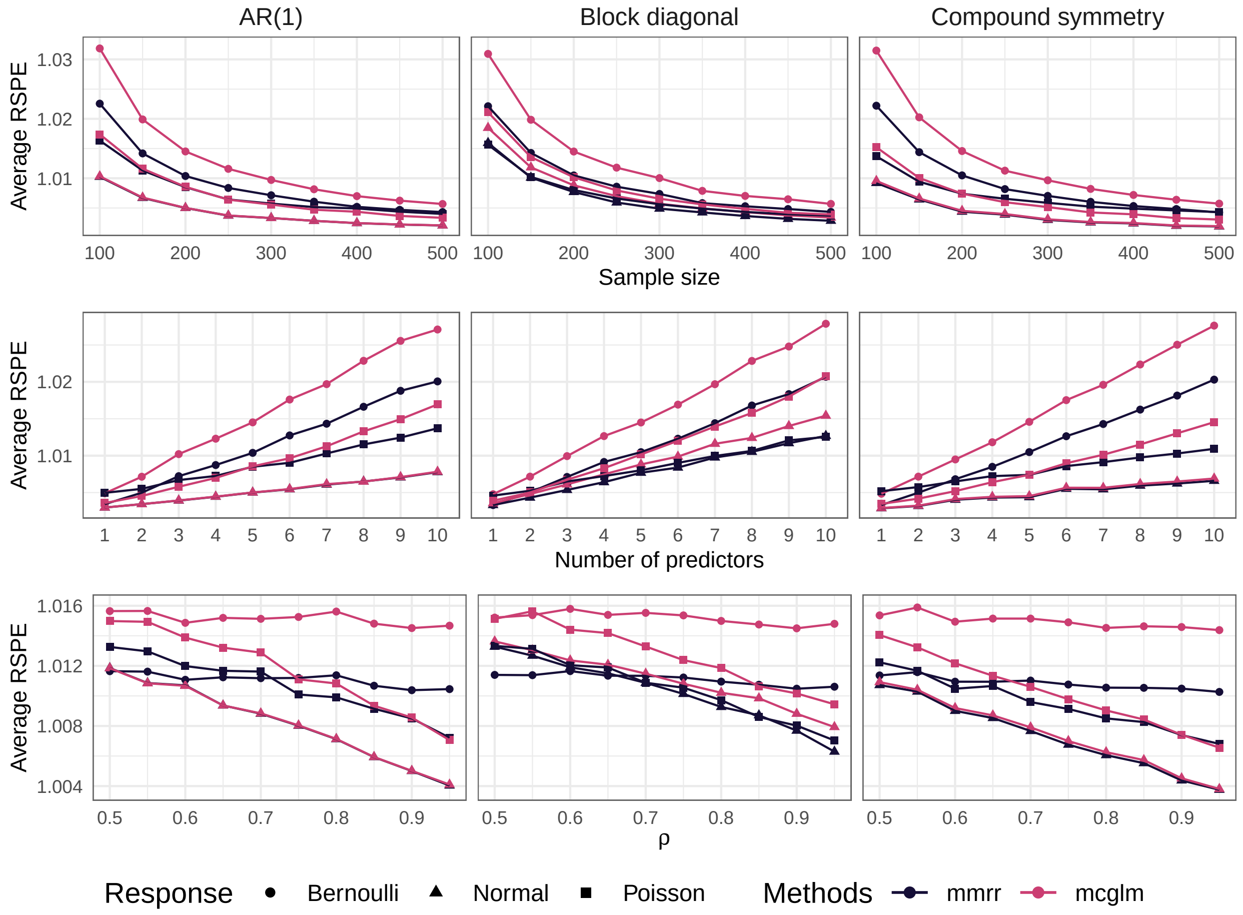

Figure A: Average relative squared prediction errors. Top: $\rho = 0.9$ and $p_j = 5$ for $j = 1, \ldots, 9$. Middle: $n = 200$ and $\rho = 0.9$. Bottom $n = 200$ and $p_j = 5$ for $j = 1, \ldots, 9$. mmrr is the proposed method and mcglm is the method of Bonat and Jørgensen (2016).

components of $Y \mid W$. In Figure E, we present average mean squared errors for estimating off-diagonal blocks of Σ using `mmrr`. The 3×3 off-diagonal black corresponding to Normal and Poisson response covariance is estimated best, whereas both blocks including Bernoulli responses are estimated more poorly. Encouragingly, we see that as the correlation parameter ρ increases, we more accurately estimate all off-diagonal blocks (e.g., see the bottom row of Figure E).

F.3 Bernoulli responses

In this subsection, we perform another simulation study to examine how our method performs with a large number of Bernoulli responses. Data are generated in the same manner as in Section 5.2, except with a single normal response and eight Bernoulli response variables. Results are displayed in Figure F. As before, we observe that as ρ increases from 0.5 to 0.95,

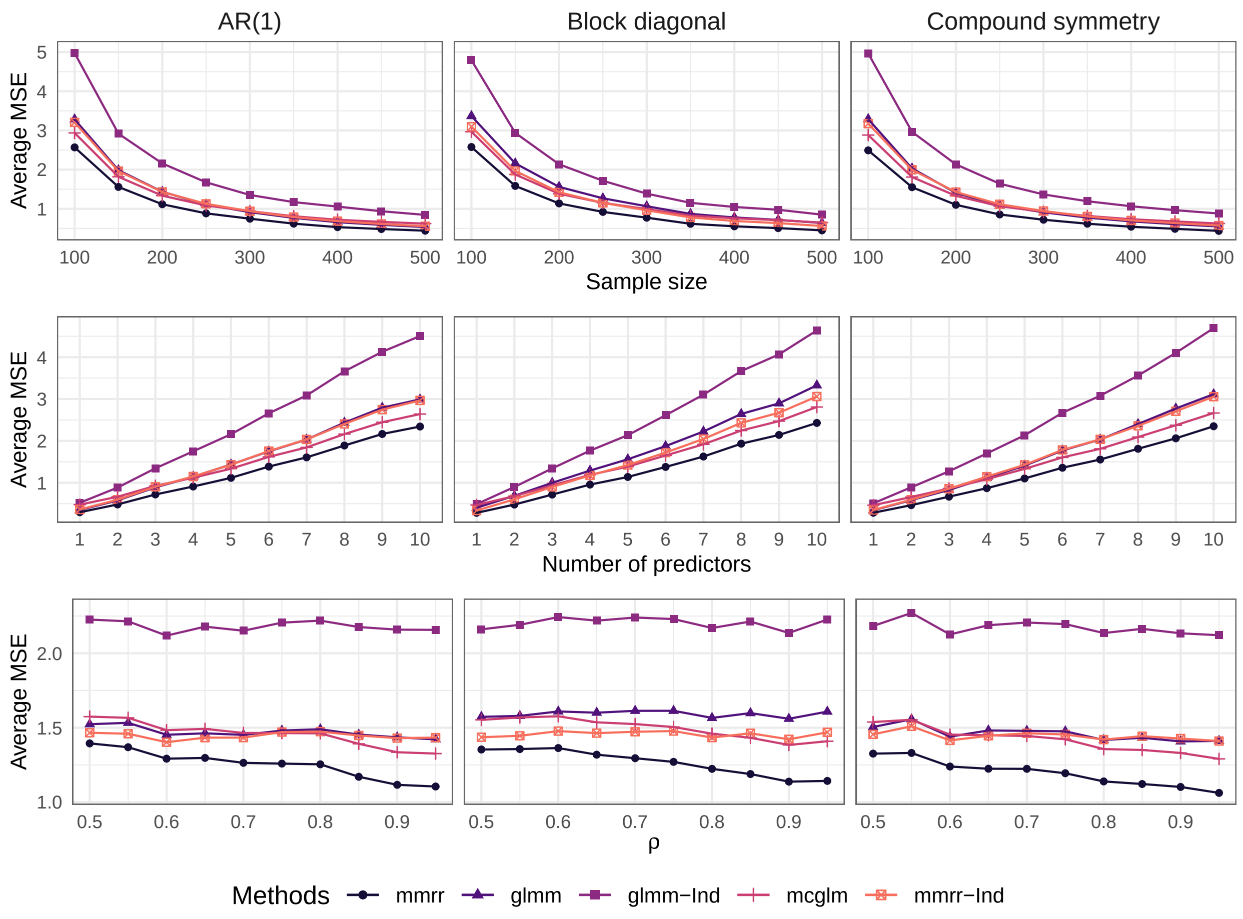

Figure B: Average mean squared estimation error of β. Top: $\rho = 0.9$ and $p_j = 5$ for $j = 1, \ldots, 9$. Middle: $n = 200$ and $\rho = 0.9$. Bottom: $n = 200$ and $p_j = 5$ for $j = 1, \ldots, 9$.

the difference between joint and separate modeling becomes more apparent. Notably, the relative mean squared prediction error for the single normal response variable improves more dramatically under both the autoregressive and compound symmetric covariance structures. Under the block diagonal covariance, the differences are less apparent. This agrees with intuition as under the block diagonal covariance structure, the normal response is only correlated with two of the Bernoulli responses, whereas with the other structures it is correlated with all eight Bernoulli responses. Together with the results in the main article, these results suggest that substantial efficiency gains can be achieved using our method for joint modeling of mixed-type responses – even in the case where most response variables are binary.

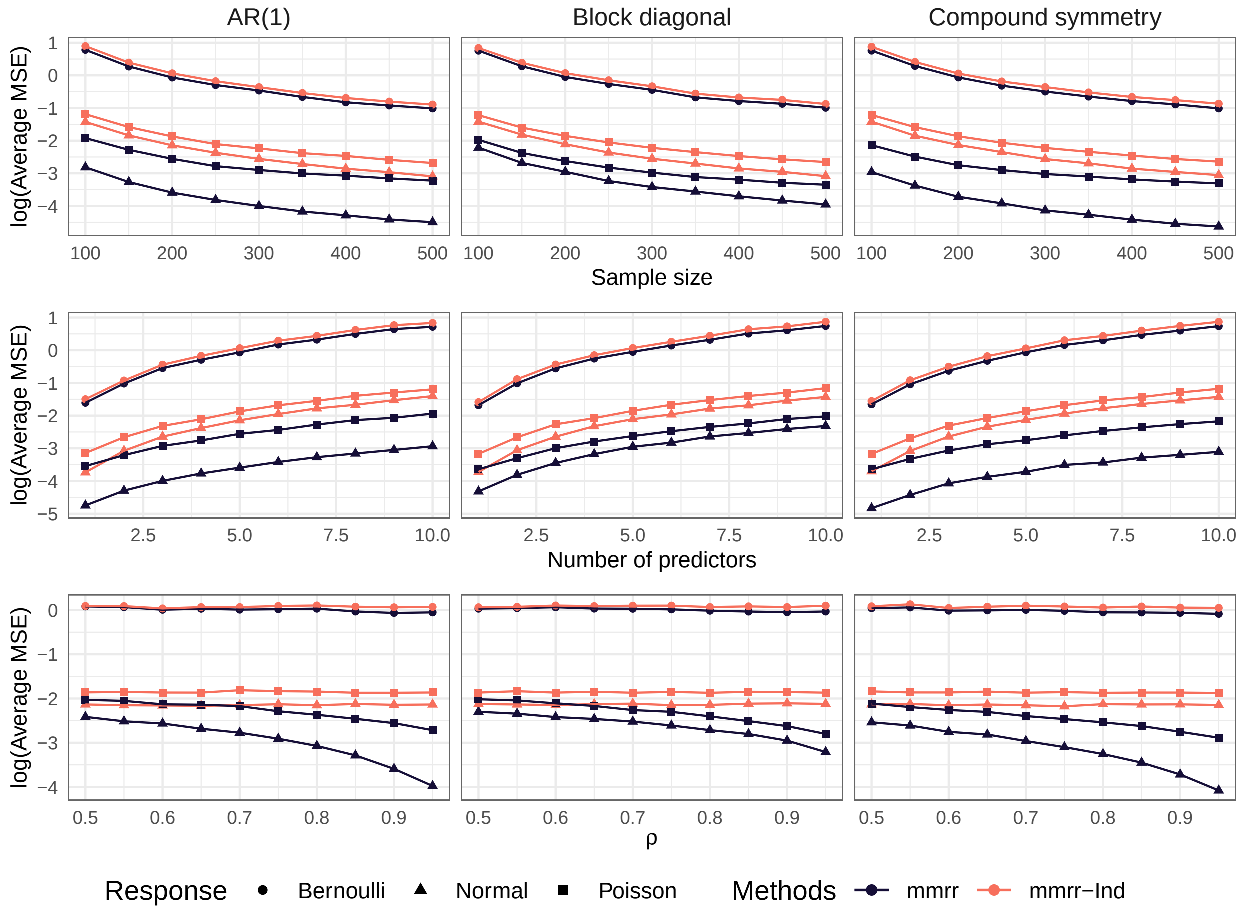

Figure C: Average mean squared estimation error (on the log-scale) of β aggregated by type. Top: $\rho = 0.9$ and $p_j = 5$ for $j = 1, \dots, 9$. Middle: $n = 200$ and $\rho = 0.9$. Bottom: $n = 200$ and $p_j = 5$ for $j = 1, \dots, 9$.

F.4 Comparison to separate GLMs

First, we present simulation study results identical to those from Figure 1 of the main manuscript, but with separate GLMs also included as a competitor. These results are displayed in Figure G. Separate GLMs perform substantially worse than all other competitors in every scenario considered. The performances of the alternative methods, though indistinguishable here, are exactly as in Figure 1 of the main manuscript.

G Osteoarthritis initiative data analysis

In this section, we analyze data collected through the Osteoarthritis Initiative (OAI), a prospective observational study of knee osteoarthritis progression (`nda.nih.gov/oai/`). Following McCulloch (2008), who kindly shared the data, we model two outcome variables:

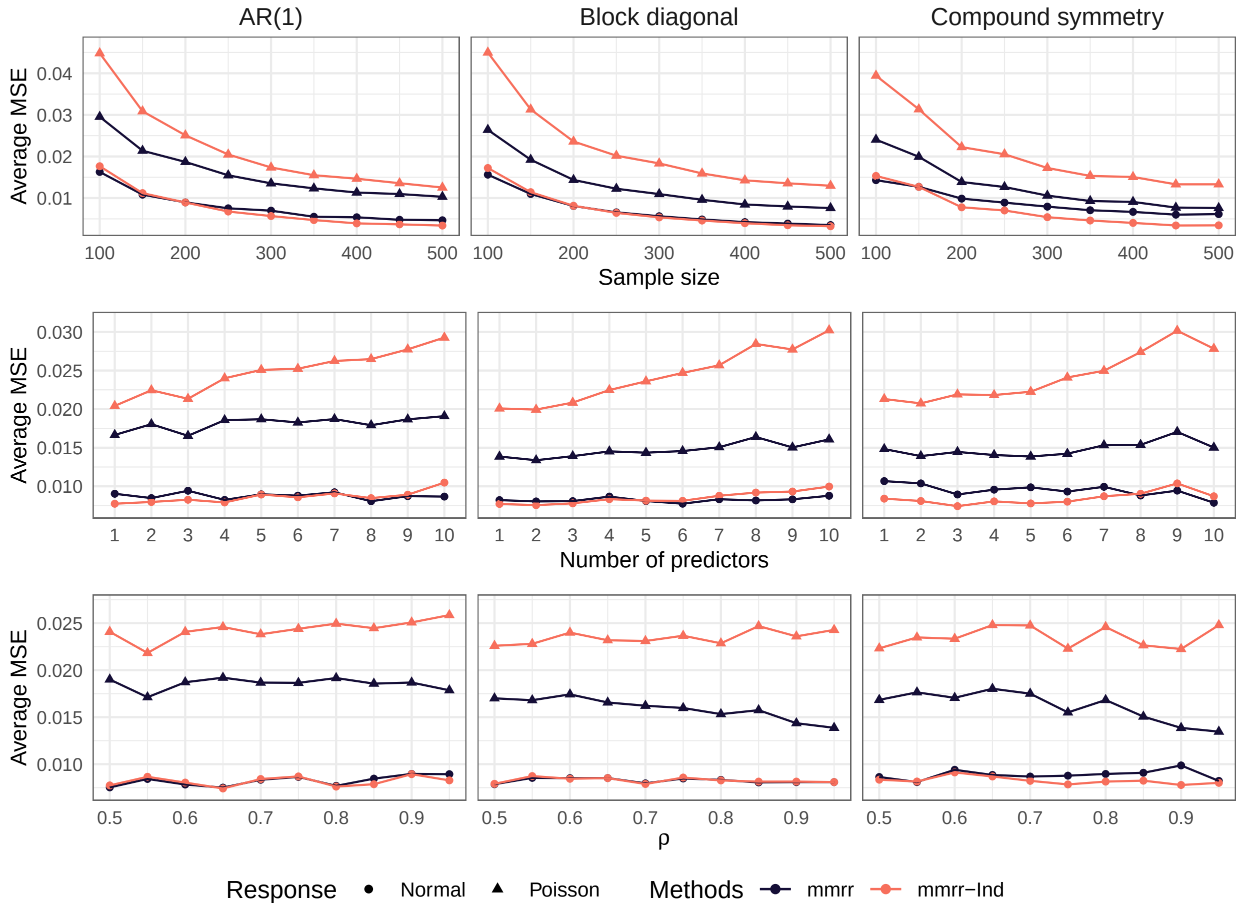

Figure D: Average mean squared estimation error for the 1st–3rd (normal) and 7th–9th (Poisson) diagonal elements of Σ. Top: $\rho = 0.9$ and $p_j = 5$ for $j = 1, \ldots, 9$. Middle: $n = 200$ and $\rho = 0.9$. Bottom: $n = 200$ and $p_j = 5$ for $j = 1, \ldots, 9$.

Western Ontario and McMaster Univerities disability score (WOMAC), and the number of days of work missed in the three months proceeding data collection. The WOMAC scores are modelled as a normal random variable after adding one and performing a log-transformation; whereas the number of days of work missed are treated as quasi-Poisson random variables. To model these data, we consider BMI, age, and sex as predictors. As in the fertility data analysis, we set $\psi_j = 10^{-2}$ for the normally distributed response and $\psi_j = 10^{-1}$ for the quasi-Poisson response. The goal of our analysis was to test for the effect of each of the three predictors on both responses simultaneously. Our analysis included only those subjects who had no missingness in either response variables or predictors, so that $n = 1602$. Fitting the full model to the data, we obtain the coefficient estimates listed in Table B. Based on the results, we would conclude that both BMI and Sex are significant predictors for both response variables, while Age did not reach the .05 significance cutoff.

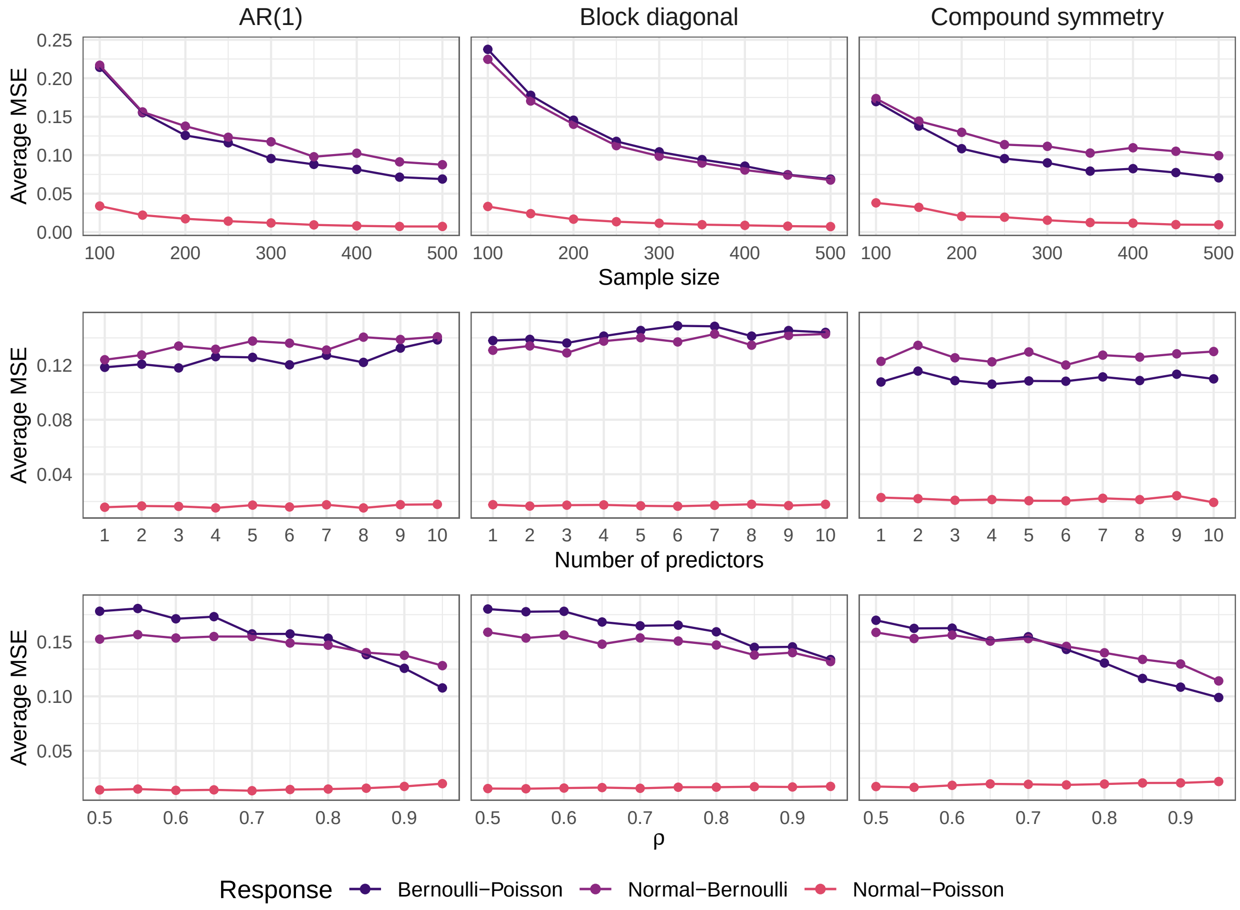

Figure E: Average mean squared estimation error for off-diagonal blocks of Σ. Top: $\rho = 0.9$ and $p_j = 5$ for $j = 1, \ldots, 9$. Middle: $n = 200$ and $\rho = 0.9$. Bottom: $n = 200$ and $p_j = 5$ for $j = 1, \ldots, 9$.

References

Bates, D., Mächler, M., Bolker, B., and Walker, S. (2015). Fitting linear mixed-effects models using lme4. *Journal of Statistical Software*, 67(1).

Bonat, W. H. and Jørgensen, B. (2016). Multivariate covariance generalized linear models. *Journal of the Royal Statistical Society: Series C (Applied Statistics)*, 65(5):649–675.

Brooks, M. E., Kristensen, K., van Benthem, K. J., Magnusson, A., Berg, C. W., Nielsen, A., Skaug, H. J., Mächler, M., and Bolker, B. M. (2017). glmmTMB balances speed and flexibility among packages for zero-inflated generalized linear mixed modeling. *The R Journal*, 9(2):378–400.

Knudson, C., Benson, S., Geyer, C., and Jones, G. (2021). Likelihood-based inference for generalized linear mixed models: Inference with the R package glmm. *Stat*, 10(1):e339.

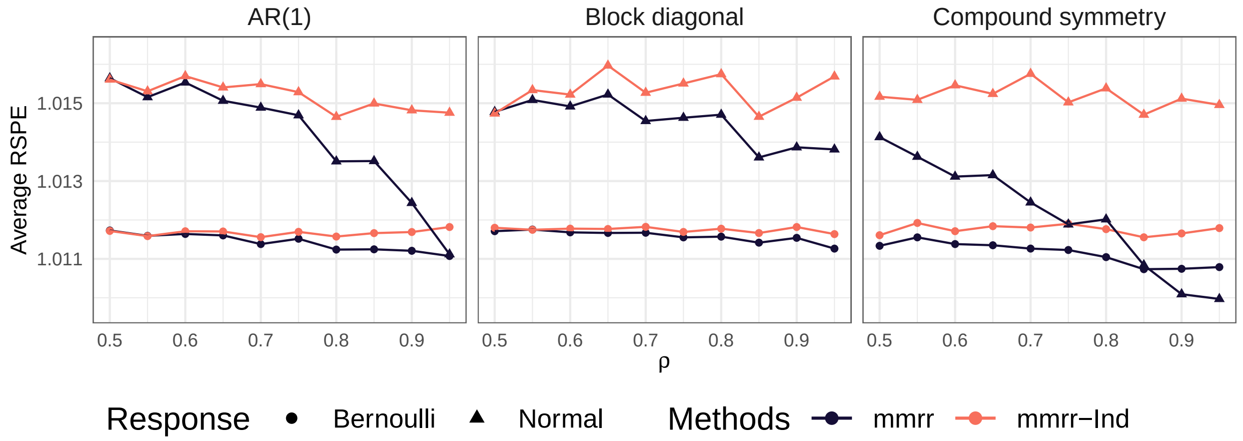

Figure F: Average relative squared prediction errors over 500 independent replications as the correlation parameter ρ varies with $n = 200$ and $p_j = 5$ for $j = 1, 2, \ldots, 9$.

Coefficient	WOMAC score	Days missed	p-value
Intercept	-0.23822	-4.67790	
BMI	0.02885	0.15758	4.155×10^{-26}
Age	0.00172	-0.03269	2.855×10^{-1}
Sex	0.13314	-0.31994	6.948×10^{-6}

Table B: Regression coefficient estimates (i.e., $\hat{\mathcal{B}}$) for the three predictors and two response variables in the OAI data analysis. In the rightmost column is the p-value for the test that the corresponding row of $\mathcal{B}$ is entirely zero.

McCulloch, C. E. (2008). Joint modelling of mixed outcome types using latent variables. *Statistical Methods in Medical Research*, 17(1):53–73.

Schabenberger, O. (2005). Introducing the GLIMMIX procedure for generalized linear mixed models. *SUGI 30 Proceedings*, 196.

Venables, W. N. and Ripley, B. D. (2002). *Modern applied statistics with S*. Springer, New York, fourth edition.

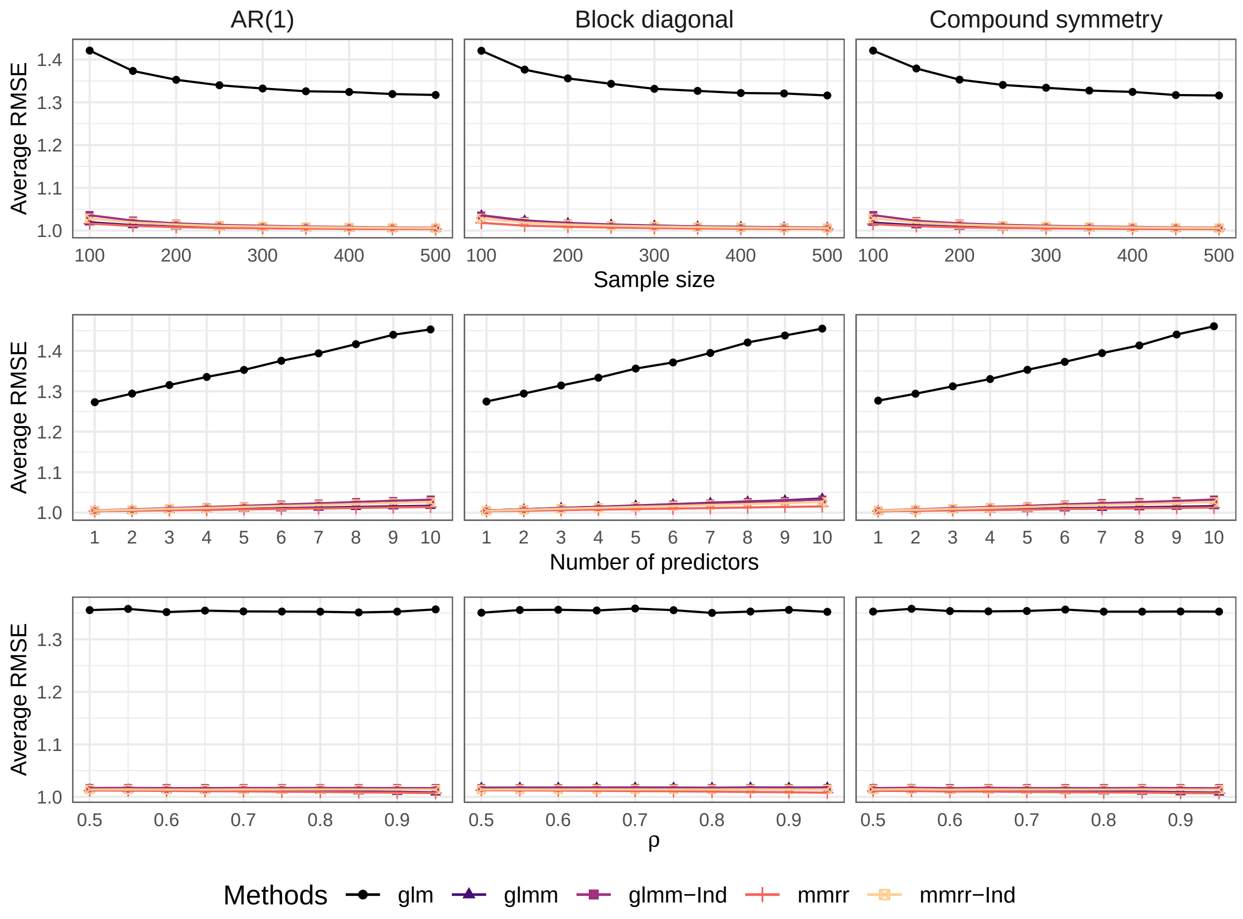

Figure G: Average relative squared prediction errors. Top: $\rho = 0.9$ and $p_j = 5$ for $j = 1, \ldots, 9$. Middle: $n = 200$ and $\rho = 0.9$. Bottom: $n = 200$ and $p_j = 5$ for $j = 1, \ldots, 9$.
